# Reuse potential of insulating glass units based on in-situ measurements and comparative calculations

**DOI:** 10.1007/s40940-026-00329-3

**Published:** 2026-06-27

**Authors:** Michael Peter Mayrhofer, Philipp Kiesslich, Andreas Taras, Vlad-Alexandru Silvestru

**Affiliations:** Institute of Structural Engineering, ETH Zurich, Zurich, Switzerland

**Keywords:** Glass reuse, Circular construction, In-situ measurements, Functional performance, Structural performance

## Abstract

Assessing the potential for reusing glass from existing windows and façades requires a systematic understanding of the systems, glazing assemblies, glass types, and glass dimensions that were applied over the years, as well as the requirements these systems had to fulfil. Existing façade and window systems largely determine the feasibility and effort required for disassembly, as well as the possibility to recover reusable or recyclable components or materials. Glazing assemblies and glass types from end-of-life façades and windows might limit the possibilities for further processing, while element dimensions might impose constraints on future façade designs based on reuse. This paper presents a systematic investigation of the glazing from nine educational and research buildings with metal-framed façades or windows, constructed or refurbished between 1967 and 2013. The available amount of glass was documented with respect to number, dimensions, assembly, and layer thicknesses. In-situ measurements of thermal and light transmittance were conducted and compared with software-based calculations. Furthermore, the structural performance of selected glazing assemblies was evaluated in relation to current design standards. The results provide a representative basis for assessing the reuse potential of existing glazing in metal-framed façades and identify key characteristics and challenges expected to influence disassembly, separation processes, and future circular façade design strategies.

## Introduction

The building sector is responsible for nearly 40% of global greenhouse gas emissions (European Commission 2020), and hence, it plays a critical role in achieving climate targets like limiting global warming according to the Paris Agreement (United Nations 2015). While past research and regulations have primarily focused on reducing operational carbon emissions, embodied carbon associated with material production, construction, maintenance, and end-of-life processes has become increasingly significant (Gibbons and Orr 2022). Glass is theoretically a highly recyclable material; however, developments aimed at improving thermal, acoustic, and safety performance have led to increasingly complex glazing assemblies. Modern insulating glass units (IGUs) often consist of coated and laminated glass panes combined with spacer systems and sealants, which significantly complicates separation processes and limits the recovery of uncontaminated flat glass suitable for high-quality recycling (Rose et al. 2019). Although recycled flat glass cullet can reduce energy demand and CO₂ emissions during glass production, current flat glass manufacturing predominantly relies on pre-consumer cullet because of contamination concerns associated with post-consumer glass (Hartwell et al. 2022). Recent pilot projects and research initiatives demonstrate that façade glass recycling is technically feasible when careful dismantling and sorting strategies are applied (Saint-Gobain Glass 2023; Tishman Speyer 2018), yet large-scale implementation remains limited.

From a climate mitigation perspective, reusing or remanufacturing glazing assemblies is more effective than recycling, as it avoids energy-intensive remelting processes (Allwood et al. 2011). Despite growing interest in glass reuse within research and industry (DeBrincat and Babic 2018; Hartwell and Overend 2019), significant challenges remain. These include uncertainties regarding the condition of existing glazing, variability in glass assemblies, unknown performance degradation over time, and compliance with current thermal and structural requirements. Moreover, systematic knowledge about the quantities, dimensions, configurations, and performance of glazing systems currently in use is scarce (Augiseau and Barles 2017).

A study on circular valorization strategies for façade glass in Belgium pointing out related barriers and opportunities was conducted by (Geboes et al. 2023). The results indicated that limited collaboration between actors, challenging logistics, time and cost pressure for demolition, and the uncertain quality and performance of the IGUs at their end-of-life are the main barriers for implementing circular approaches for aged IGUs. Studies on the life-expectancy of IGUs and factors influencing their condition were already conducted in the 1990s by (Wolf 1992; Wolf and Waters 1993) and emphasized the importance of a gas-tight edge seal. However, over the years various IGU spacer and edge seal systems were developed, mostly to improve the thermal properties of the IGUs. An overview is provided by (Van Den Bergh et al. 2013).

The potential of glass reuse can also be influenced by the different façade and window systems used in the past. The necessary effort, time and cost for disassembly varies for different façade systems (e.g., punched window façade compared to post-and-beam façade) and can determine whether a consequent material separation is considered feasible or not by building owners and demolition companies. Investigations on different window systems, their condition and their reuse potential were conducted for example by (Snitzel et al. 2025). Glass types (such as monolithic versus laminated glass, annealed versus thermally tempered glass, and coated versus uncoated panes) further constrain possible reuse and remanufacturing pathways, as tempered glass cannot be cut or drilled and coatings may limit subsequent processing. In addition, the dimensions of recovered glass elements impose constraints on future façade designs and remanufacturing strategies. Knowing these characteristics for the end-of-life glass becoming available in the near future because of refurbishments of façades or replacements of buildings, and understanding related limitations is essential for enabling circular façade design strategies. While for older façades, assessing these characteristics requires in-situ measurements and is labor-intensive, for more recent and future façades, emerging tools such as material passports (Çetin et al. 2023; Honic et al. 2021) will significantly simplify the access to relevant data, including façade systems, IGU assemblies, dimensions, quantities, and physical performance parameters such as thermal and optical properties.

With more and more ageing buildings and façades, condition assessment investigations gained importance in recent years. Previous studies focused mainly on assessing the thermal performance of IGUs. Several investigations were conducted in the past decade based on in-situ measurements of the thermal transmittance, as for example (Feng et al. 2020), (Park et al. 2021), or (Likins-White et al. 2023). A review on different methods for in-situ thermal transmittance measurements is provided by (Soares et al. 2019). Others focused on argon gas concentration in the cavity as an assessment method for the condition of the IGUs, and their reuse potential, like (Niiranen et al. 2018) and (van Nieuwenhuijzen et al. 2023). Moreover, (Davis et al. 2025) analyzed besides gas content reduction over the years, also a potential degradation of the coatings, based on emissivity measurements. (Teich et al. 2024) performed gas content measurements and dew point temperature measurements to assess the cavity tightness of aged IGUs. Despite recent advances in material flow analysis and condition assessment of end-of-life glazing (Rota et al. 2023), comprehensive empirical studies combining documentation-based analysis, in-situ performance measurements, software-based calculations, and structural verification for real building stocks remain limited. In particular, there is a lack of case studies assessing existing IGUs with respect to their reuse potential both under current thermal and structural standards. While for efficiently implementing reuse and remanufacturing of IGUs and glass in the façade industry, the required processes for quality assessment need to be minimized, collecting extensive data on the condition of aged glass and analyzing the significance of different characteristics on the reuse and remanufacturing potential is essential.

This paper addresses this gap by presenting a systematic investigation of glazing assemblies from nine educational and research buildings with metal-framed façades located in Zurich. The buildings were constructed or refurbished between 1967 and 2013 and represent a range of different façade typologies and glazing configurations. The study combines (*i*) a documentation- and field-study-based analysis of glazing quantities, dimensions and types, (*ii*) in-situ measurements of thermal and optical performance, (*iii*) software-based functional performance calculations, and (*iv*) structural assessments according to current standards. The results provide a comprehensive basis for evaluating the reuse and remanufacturing potential of existing glazing and contribute to the development of circular strategies for glazed façade systems.

## Methods

The study included nine buildings located in Zurich, whose façades were constructed or renovated between 1967 and 2013. The selection of buildings was based on a balanced temporal distribution across several decades, with particular emphasis on older buildings, as well as on accessibility for on-site investigations. One of the nine buildings (building B5) underwent a later extension, while the original building part was renovated at a different point in time. Due to the resulting differences in façade construction periods, the two building parts were treated as separate case-study buildings in the analysis (building B5a and B5b).

The subsequent analysis showed that the investigated buildings encompass a broad range of façade systems and glazing assemblies. The observed glazing assemblies included monolithic glazing, double and triple insulating glass units (IGUs), as well as IGUs with and without coatings.

**Fig. 1:**
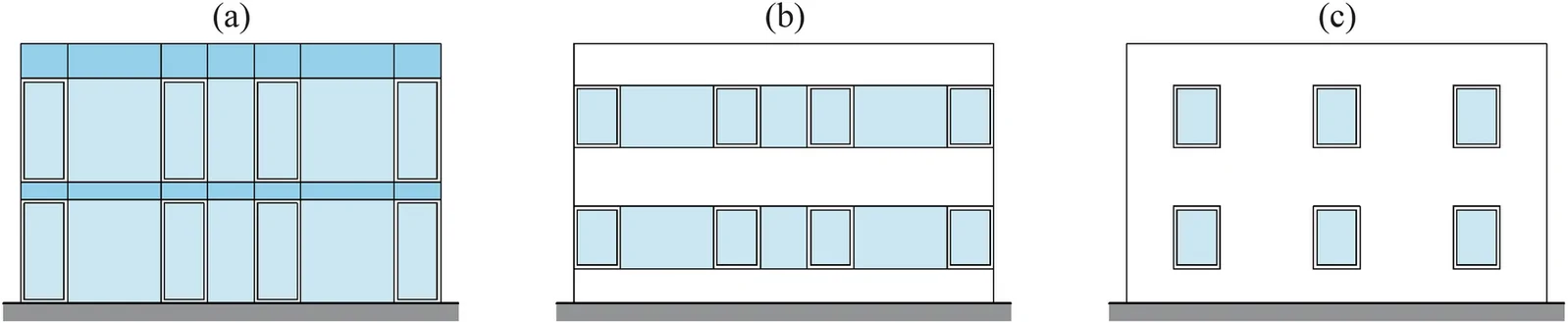
Schematic overview of the façade typologies identified in the investigated buildings: (**a**) post-and-beam façade, (**b**) ribbon glazing façade, and (**c**) punched window façade

Based on their architectural and constructional characteristics, three principal façade typologies were identified: post-and-beam façades (Fig. 1a), ribbon glazing façades (Fig. 1b), and punched window façades (Fig. 1c). These typologies differ in terms of structural concept, glazing subdivision, and integration of glazing within the façade, resulting in distinct boundary conditions with respect to disassembly strategies and potential reuse applications.

One investigated building was excluded from the further analysis, as its façades predominantly consisted of enameled fully tempered glass (FTG). Due to the material characteristics of enameled glass, these glazing units were considered unsuitable for reuse, are likely also unsuitable for recycling, and were therefore not included in the assessment.

**Fig. 2:**
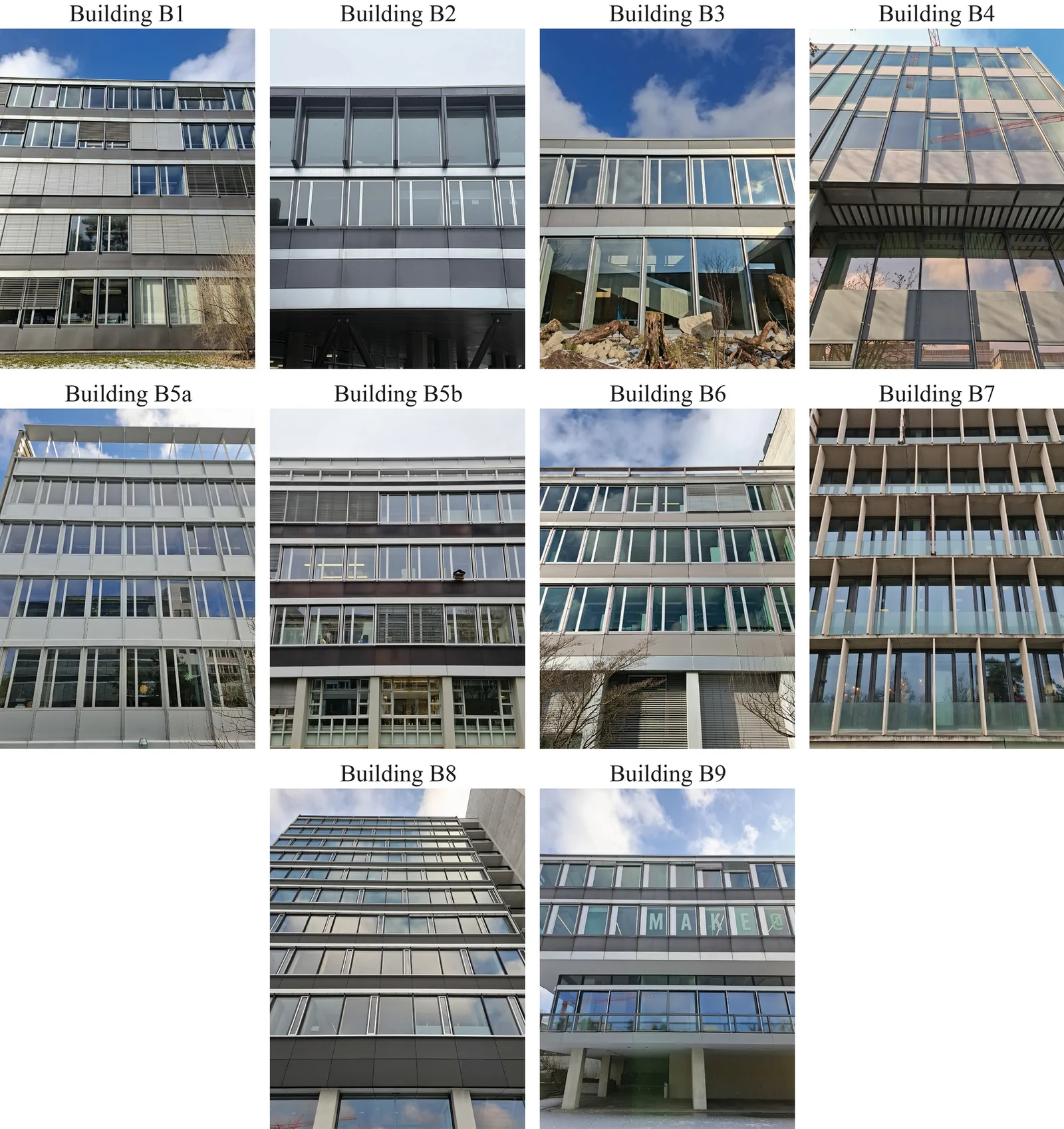
Representative views of the investigated façades from the nine different buildings, illustrating the relevant glazed façade areas considered in this study

An overview of the glazed façade areas by façade type and the corresponding glazing weights for all investigated buildings is provided in Table 1. Representative views of the investigated façades from the nine different buildings, illustrating the relevant glazed façade areas considered in this study are shown in Fig. 2.

**Table 1:** Overview of glazed façade areas differentiated by transparent façade typology and glazing weight for the different investigated buildings

Building	Façade construction year	Post-and-beam façade area (m^2^)	Ribbon glazing façade area (m^2^)	Punched window façade area (m^2^)	Other glazed façade area^a^ (m^2^)	Total glazed façade area (m^2^)	Glazing weight (t)
B1	1967	218.0	0	1026.8	0	1244.7	25.8
B2	1973	397.2	0	598.0	0	995.1	32.5
B3	1973	11.7	0	419.6	0	431.3	11.2
B4	1976	8160.9	0	0	0	8160.9	285.6
B5a	1995	51.3	0	398.0	0	449.3	9.5
B5b	2002	43.1	90.6	632.4	0	766.0	18.2
B6	2002	58.6	0	504.6	108.7	671.8	21.1
B7	2008	119.3	2777.3	0	0	2896.6	144.8
B8	2012	399.0	1588.9	149.2	0	2137.1	111.3
B9	2013	189.6	318.7	0	0	508.3	18.9

^a^Other glazed façade area includes all remaining façade types that could not be assigned to the other three categories. For building B6, this area consists of IGUs mounted to the steel substructure using point fixings. This façade area is not illustrated in the representative views of the investigated façades, as it represents only a minor share of the total glazed façade area

### Analysis of glass dimensions and quantities

To determine the dimensions, quantities, IGU assemblies, as well as the façade systems, available building documentation was analyzed for each building. It was observed that documentation was often incomplete, particularly for older and smaller buildings. In contrast, the availability and level of detail of documentation increased significantly for newer buildings and for projects with higher investment volumes. For almost all buildings, relevant floor plans, building sections, and façade elevations could be identified. Detailed façade sections and construction details, however, were generally only available for the newer and larger buildings.

Information on IGU dimensions obtained from drawings, sections, and details was subsequently verified on site. This verification was carried out in a targeted manner for selected glazing units and façade areas and served to confirm the reliability of the documented information. This preliminary analysis of the documentation allowed the IGUs requiring on-site measurements to be identified in advance, thereby significantly reducing the time required for the more labor-intensive field measurements and forming the basis for the subsequent investigations described in Sects. 2.2–2.5.

### Measurements of glazing assembly

**Fig. 3:**
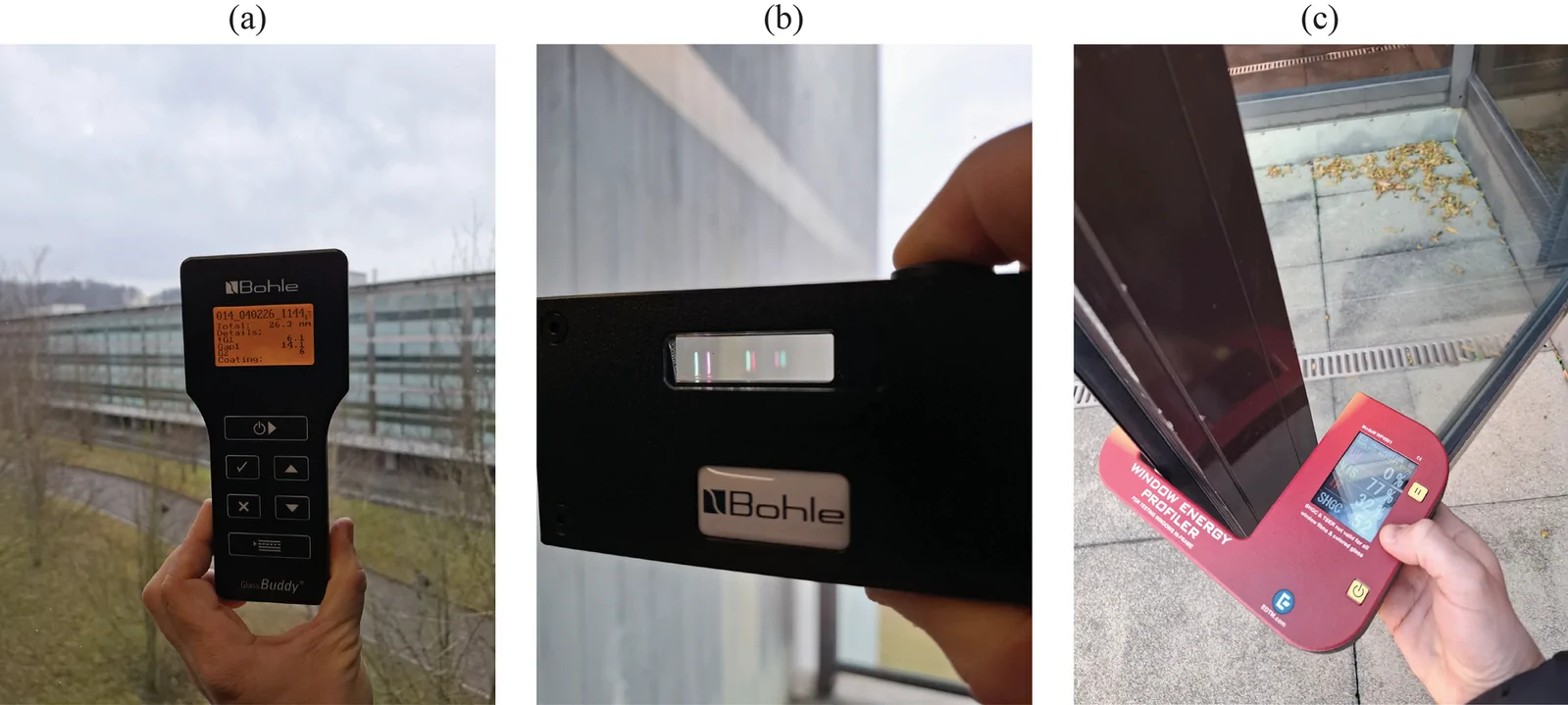
Measurement equipment used for the in-situ assessment of glazing assemblies and functional performance: (**a**) measurement of IGU configuration using the GlassBuddy® Plus device, (**b**) non-destructive identification of thermally toughened glass using the Merlin TGI—Toughened Glass Indicator, and (**c**) measurement of optical properties using the Window Energy Profiler WP4501

The configuration of the IGUs was determined to identify the number and thickness of panes, the presence of laminated glass, and the use of coatings. Measurements were performed using the GlassBuddy® Plus device from Bohle AG, which allows the non-destructive assessment of monolithic glass, as well as double and triple IGUs. The instrument also provides information about the existence of laminated glass (LG) and coated glass within the assemblies, and specifies the thickness of the different glass layers and interlayers. The measurements were conducted on representative and accessible IGUs identified during the documentation analysis described in Sect. 2.1. A measurement using the GlassBuddy® Plus device is illustrated in Fig. 3a. Several measurements were conducted on each glazing to ensure correctness and consistency of the determined data. Moreover, the measurements were performed relatively close to the framing to ensure that a potentially existing deformation due to climate loads was not influencing the measurement.

For building no. B4 the determination of the IGU configuration was significantly impeded by a highly reflective coating, which limited the reliability of the device readings. In this case, information obtained from available façade sections was used and cross-checked with data from an IGU that had previously been replaced for maintenance purposes. The limited measurement results obtained using the GlassBuddy® Plus were therefore considered indicative only and were supplemented by documentation evidence.

The determination of the glass type (annealed or thermally toughened) was initially based on the evaluation of available documentation and a visual inspection of the glass panes, with particular attention paid to the presence of ceramic stamps applied during the thermal toughening process. To verify these assessments, measurements were subsequently carried out using the Merlin TGI—Toughened Glass Indicator from Bohle AG, which allows a non-destructive evaluation of whether a glass pane has been thermally toughened or not. To assess whether the glass has been thermally toughened, the device was moved across the glass surface while the interference lines visible in the viewing window were observed. These lines represent the different surfaces of the glass packages in the IGU. A constant color of the lines indicated non-toughened glass, whereas a blurry color change of one of the lines when moving the device indicated thermally toughened glass. A measurement using the Merlin TGI device is illustrated in Fig. 3b. It is worth noting that the measurements also worked well for coated glass, since the color of the visible lines was different, but did not change when moving the device across the glass surface. To ensure reliability of the assessments, several readings were performed for each assessed glazing, and whenever possible, measurements from both IGU sides were performed.

### Light transmittance measurements

Light transmittance measurements were conducted to characterize the optical performance of the IGUs in-situ. Measurements were performed using the Window Energy Profiler WP4501 from EDTM, Inc., a device designed to assess the energy-related properties of installed windows. The instrument features an opening sufficiently large to be positioned over the sash of an in-situ openable window. The WP4501 provides four real-time measurement outputs: the estimated solar heat gain coefficient (SHGC), as well as ultraviolet (UV), visible light (VL), and infrared (IR) transmission values. Measurements were performed in-situ and provided real-time values of the optical properties. The obtained results were interpreted as indicative in-situ measurements and were primarily used for comparative analysis between different IGU configurations rather than for absolute performance certification. A measurement using the Window Energy Profiler WP4501 device is illustrated in Fig. 3c.

### Thermal transmittance (U-Value)

Previous studies have demonstrated that the in-situ determination of the thermal transmittance of glazing systems using heat-flux and temperature measurements provided reliable results when steady-state conditions in accordance with (ISO 9869-1, 2014) were fulfilled, see also (Paschke et al. 2021). These conditions require that heat-flux meters and temperature sensors are installed away from thermal bridges, cracks, or other local sources of disturbance, and are not exposed to direct influences from heating or cooling devices or air draughts. In addition, the external surface of the investigated element must be protected from rain, snow, and direct solar radiation, where necessary, by means of temporary artificial screening.

**Fig. 4:**
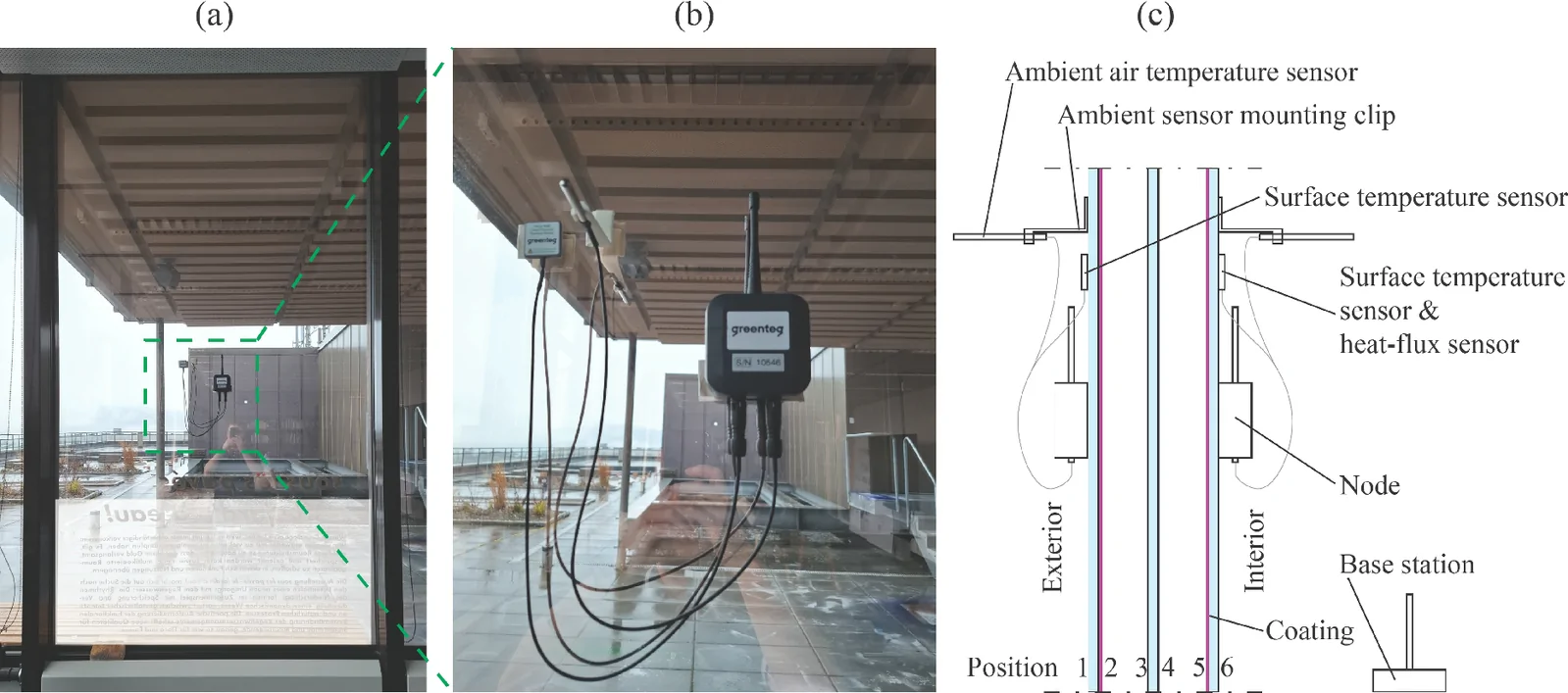
In-situ measurement setup of the gOMS II (UVAL Wireless System): (**a**) overview of an investigated IGU showing the positions of the installed sensors, (**b**) close-up view of the mounted ambient temperature, surface temperature, and heat-flux sensors, and (**c**) schematic section of the IGU illustrating the sensor locations, the numbering of glass surfaces in an IGU, and the common position of coatings within the glazing assembly of a triple IGU (red lines)

The in-situ measurements of the thermal transmittance (U-value) and thermal resistance (R-value) of the IGUs was carried out using the gOMS II (UVAL Wireless System) from greenteg AG. The measurement setup consisted of wireless sensor nodes installed on both the interior and exterior sides of the glazing, which continuously transmitted the recorded data to a central base station. The installed sensor nodes included ambient air temperature sensors on the interior and exterior sides, an exterior surface temperature sensor, and an interior surface temperature and heat-flux sensor (see Fig. 4c).

Air temperature sensors were installed using the supplied ambient sensor mounting clips to ensure sufficient distance from the glazing surface, thereby avoiding measurement errors caused by the lower surface temperature of the glass. Surface temperature sensors were mounted exclusively using the supplied double-sided adhesive tapes, while the exterior sensor, where required, was installed using water-repellent double-sided adhesive strips. To minimize edge effects and local disturbances, all surface and heat-flux sensors were positioned as centrally as possible on the glazing units (see example in Fig. 4a). According to (ISO 9869-1, 2014), the external surface temperature sensor shall be mounted on the external surface opposite the heat-flux sensor as illustrated in Fig. 4b. According to the gOMS II User Manual, sensor accuracy is ensured only when greenteg-approved mounting solutions are used. The use of non-approved mounting solutions may result in measurement errors and could potentially lead to safety issues. The measurement interval was set to one minute.

For each building, the U-value and R-value were measured for one of the most frequently occurring glass assemblies. To account for variability within the same glazing type, four different IGUs of the selected configuration were measured per building under comparable boundary conditions.

According to the gOMS II User Manual, a sufficiently large temperature difference between the interior and exterior environments (ΔT ≥ 5 °C) was required to obtain meaningful heat-flux measurements. Consequently, measurements could not be conducted during summer. All measurements were therefore carried out between mid-September and the end of January, with particular attention paid during September and October to restrict measurements to nights with a sufficiently large temperature difference. Measurements were thus limited to periods with relatively stable external conditions.

**Fig. 5:**
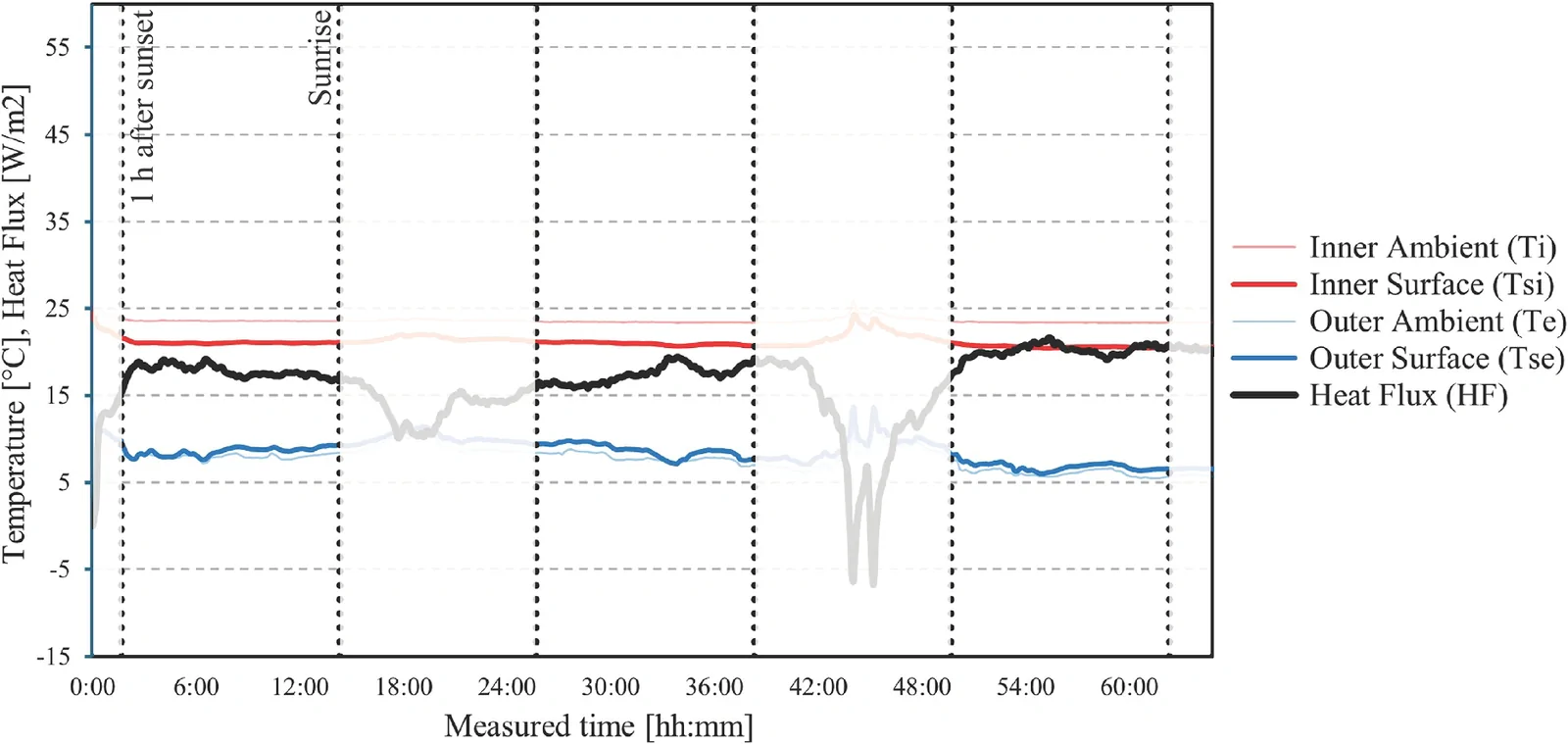
Example of an in-situ U-value and R-value measurement showing the recorded interior and exterior surface temperatures, ambient air temperatures, and heat flux over three consecutive nights. Measurement data recorded during daytime are shown with reduced opacity, as they were excluded from the evaluation

For data evaluation, only measurement results recorded during night-time conditions were considered, defined as the period from one hour after sunset until sunrise. This restriction was applied because solar radiation can cause the exterior glass pane to warm up more rapidly than the inner panes, which may lead to a temporary reversal of the heat-flux direction despite lower exterior air temperatures. Including such periods in the evaluation would introduce errors in the calculated thermal resistance. Measurements were therefore terminated once the calculated R-values exhibited variations of no more than ± 5% over three consecutive nights, ensuring convergence of the results. An example of the measured surface temperatures, ambient air temperatures, and heat flux over three consecutive nights is shown in Fig. 5, highlighting the data used for the R-value evaluation. It can be noted that the heat flux was relatively constant during the nights, but significantly changed during the days.

A major practical challenge was the presence of automatically operated external blinds. To avoid any influence on the measurements and to protect the measurement equipment, these shading systems were fully deactivated for the entire measurement period. In several cases, this required disabling the shading systems for the whole building, which significantly constrained scheduling under ongoing building operation. Further constraints arose from the need to access suitable interior spaces adjacent to the façade elements under investigation. Whenever possible, unused or rarely occupied rooms were selected to minimize disturbances caused by intermittent heating, ventilation, or occupant behavior.

In older buildings, an additional difficulty consisted in identifying glazing units that had not been replaced during the service life of the façade. This limitation complicated the accurate determination of the IGU age. To mitigate this uncertainty, supplementary assessments were performed, including the measurement of the IGU configuration, the identification of coatings, the evaluation of spacer codes where available, and the visual comparison of edge seals between different windows. Glazing units exhibiting differences in IGU configuration, coating characteristics, spacer codes, or edge seal appearance compared to the majority of windows within the same façade were interpreted as replacements carried out at a later stage, whereas units showing consistent characteristics across these criteria were taken as indicative of the original glazing installation period.

**Fig. 6:**
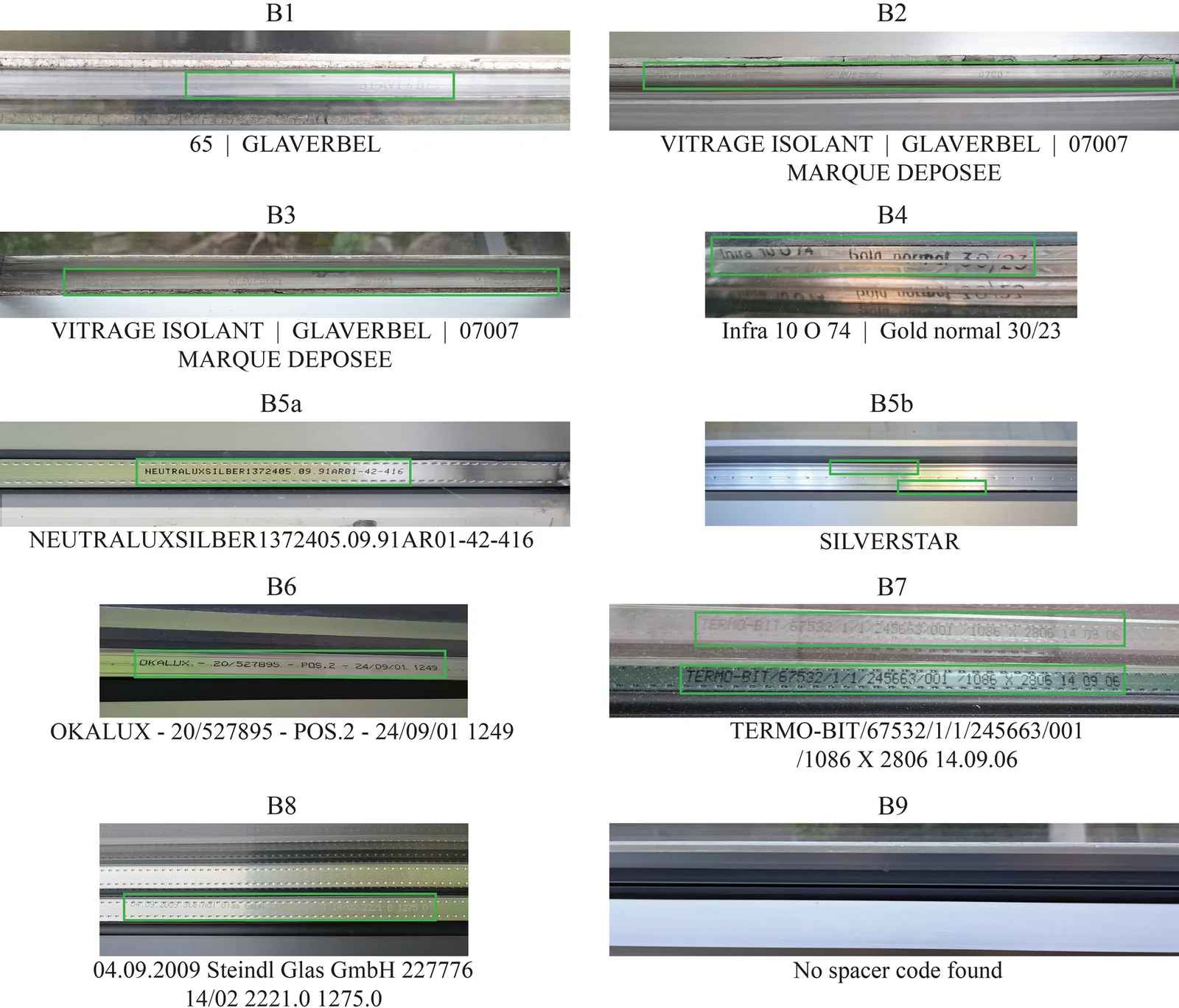
Overview of the spacers and spacer codes identified in the investigated buildings. To improve readability, the inscriptions visible on the spacers, which were partially difficult to read in the photographs, are additionally transcribed below each image

As illustrated in Fig. 6, spacers often contain additional information about the IGU. Depending on the manufacturer and production period, information such as the manufacturer, production date, coating designation, noble gas filling, glass dimensions, and further technical details may be indicated on the spacer. In some cases, additional manufacturer-specific internal codes were identified, which could potentially provide further information on the IGUs if corresponding documentation from the producer is available.

In addition to the in-situ measurements, software-based U-value calculations were performed to provide reference values for comparison and to assess potential ageing-related deviations in IGU performance. The calculations were carried out using SILVERSTAR glaCE (Glas Trösch) and the Glass Configurator (AGC Glass Europe). As historical coatings could not be identified in-situ and are likely no longer commercially available, the coatings used for the software-based calculations were selected from those available in the respective software tools to best match the measured light transmission properties. In some cases, historical coating designations were indicated on the spacer; however, determining the corresponding physical properties would require extensive additional research and would even not be possible in some cases. This would be particularly challenging when manufacturers no longer exist, have been acquired, or have changed their company name over time. As such extensive investigations are difficult to implement in practical reuse projects, a simplified and practice-oriented approach for the coating selection was adopted in the performed calculations. For this purpose, the measured and calculated UV transmission and visible light transmission values were compared, and the coating showing the closest agreement was adopted for the calculations. The infrared (IR) transmission and the estimated solar heat gain coefficient (SHGC) values were not considered for this matching process. IR transmission values cannot be directly extracted from the used software, and SHGC values derived from in-situ measurements may be unreliable when coated glass packages exhibit delayed energy release due to absorption effects. Due to incomplete documentation, the gas filling could not be determined for all IGUs. Moreover, instruments for in-situ gas content measurements were not available for these investigations. Therefore, the software-based calculations were performed assuming both 100% air-filled cavities and 90% argon-filled cavities in order to account for boundary values in terms of gas-filling scenarios. The objective of these calculations was not to determine the exact properties of the IGUs, but to assess whether simplified and practically applicable assumptions can provide reasonable estimates of their thermal performance.

To allow a direct comparison between measured and calculated thermal transmittance values without distortion by environmental influences, U-values derived from the measurements were reconstructed using the measured R-values of the IGUs in combination with standardized internal and external surface resistances.

According to (ISO 9869-1, 2014) under steady-state conditions, the thermal properties of the IGUs are defined as follows. The thermal resistance R of an element, defined surface-to-surface, is given by Eq. (1):

1 $$R=\frac{{T}_{si}-{T}_{se}}{q}$$

where ${T}_{si}$ and ${T}_{se}$ denote the interior and exterior surface temperatures, respectively, and q is the heat flux density.

The thermal transmittance U of an element, defined environment-to-environment, is given by Eq. (2):

2 $$U=\frac{q}{({T}_{i}-{T}_{e})}=\frac{1}{{R}_{T}}$$

where ${T}_{i}$ and ${T}_{e}$ represent the interior and exterior ambient air temperatures, respectively. The total thermal resistance ${R}_{T}$, is given by Eq. (3):

3 $${R}_{T}={R}_{si}+R+{R}_{se}$$

with ${R}_{si}=0.137$ and ${R}_{se}=0.04$ being the standardised internal and external surface thermal resistances according to (DIN EN 673 2025). These values are applicable for the calculation of glazing with an inclination between 60° and 90° (with 90° corresponding to a vertical glazing). The same values for ${R}_{si}$ and ${R}_{se}$ were applied consistently in both the reconstruction of U-values from the measured R-values and in the software-based U-value calculations, ensuring direct comparability between measured and calculated results.

### Structural assessment of insulating glass units

The structural assessment of the IGUs was performed to evaluate the theoretical reusability of existing glazing under current design requirements. The calculations were carried out in accordance with the Swiss standard (SIA 2057 2021) using the software MEPLA Pro (MEPLA Pro 2025). For each building, all IGUs with a higher count number than 50 were analyzed. Potential reductions in load-bearing capacity due to ageing effects were not considered in the calculations. Such aspects are part of ongoing research at different institutions (Cupać et al. 2024; Rota et al. 2023; Teich et al. 2024) and could be additionally considered in future extension of these studies. To complement this limitation, an additional evaluation based on the required minimum glass strength was carried out, allowing a more differentiated assessment of the reuse potential.

All glazing units were analyzed without horizontal live loads, as only three out of the 30 structurally assessed IGUs would potentially be subjected to live loads in the case of reuse according to (SIA 2057 2021). For all remaining cases, the application of horizontal live loads is not permitted by the current standard, which requires that, for freely accessible glazing, the risk of injury must be reduced either by appropriate constructive measures or by the selection of suitable glass types, such as fully toughened glass (FTG) or laminated safety glass (LSG). Pendulum impact tests were not considered, as none of the investigated IGUs fulfils the criteria for barrier glazing according to (SIA 2057 2021). Consequently, the investigated IGUs cannot be reused as barrier glazing, and impact resistance is therefore not a governing requirement for the reuse scenarios assessed in this study.

In addition to wind loads, climatic loads were considered in the structural assessment in accordance with (SIA 2057 2021). For the assessment of climatic loads, the influence of solar shading devices and coatings was neglected. The standard requires a local altitude difference ΔH between production altitude and installation altitude of ± 400 m to be assumed for the verification of insulating glass units. This requirement may lead to situations in which small and slender glazing units cannot be verified numerically, as the assumed pressure differences become governing. Furthermore, due to the unknown production locations of the investigated IGUs, a realistic reduction of the altitude difference could not be applied. For reuse scenarios, it must also be assumed that the pressure difference within the cavities of the IGUs is still present, as IGUs exhibiting leakage would generally be unsuitable for reuse. However, the long-term development of the pressure difference between production altitude and installation altitude over the service life of the IGUs has not yet been sufficiently investigated and remains an open research question.

For these reasons, the simplified verification according to Clause 5.4.3.8 of (SIA 2057 2021) was applied for the assessment of such glazing units. This clause states that double or triple IGUs fulfilling among others the requirements listed below may resist wind loads of up to 1.10 kN/m^2^ without further verification:

- Loads limited to wind, self-weight, and climatic loads;
- Vertically installed glazing, supported linearly along all four edges;
- Glazing area smaller or equal to 2.0 m^2^;
- Glass thicknesses between 4 and 8 mm, including LSG made of 2 × 3 mm or 2 × 4 mm panes;
- Cavity width smaller or equal to 16 mm for double IGUs or 2 × 14 mm for triple IGUs.

Similar provisions can also be found in (DIN 18008-2 2020). The following settings were applied for the simulations in the software MEPLA Pro:

- A minimum of 10 finite elements per IGU edge was applied;
- Edge support type 0 was selected to account for a hinged support;
- All rectangular glass panes were assumed to be supported linearly along all four edges;
- All glazing units were considered to be vertically installed; therefore, the self-weight load case was neglected;
- A non-linear analysis was performed to account for large deflections;
- Maximum principal stresses in the ultimate limit state (ULS) and deflections in the serviceability limit state (SLS) were checked.

To contextualize the calculated wind load capacities, reference wind loads were determined in accordance with (SIA 261 2020) for a representative reference building located in Zurich with dimensions of b = 30 m, l = 30 m, and h = 15 m. For the determination of the reference wind loads, the reference building was assumed to be located in exposure class III. This comparison allowed an assessment of which IGUs would still be suitable for use under current wind loading conditions at the same locations.

In addition, for all IGUs for which the simplified approach according to Clause 5.4.3.8 of (SIA 2057 2021) did not apply, the minimum required design glass strength (${f}_{g,d,req}$) for annealed glass (ANG) was calculated. The analysis was limited to ANG, as ANG can withstand significantly lower stresses compared to thermally toughened glass, and therefore represents the more critical case for reuse assessment. The corresponding required characteristic glass strength (${f}_{g,k,req}$), based on the governing load case and the respective glazing geometry, is given by Eq. (4). To express the results on a characteristic level, different values of the load duration factor ${k}_{mod}$ were applied depending on the governing action. For load cases governed by wind, a value of ${k}_{mod}=0.9$ was used, whereas for cases governed by climatic loads (without wind), ${k}_{mod}=0.45$ was applied.

4 $${f}_{g,k,req}=\frac{{f}_{g,d,req}*{\gamma}_{M}}{{k}_{mod}*{k}_{E}*{k}_{V}*{k}_{c}}$$

With ${\gamma}_{M}=1.8$, ${k}_{E}=1.0$, ${k}_{V}=1.0$ (${k}_{V}=1.1$ for LSG) and ${k}_{c}=1.4$.

## Results and discussion

This section presents and discusses the results of the different investigations conducted for the IGUs in the selected façades and windows. The results address IGU and glass typologies, dimensions and amounts, the comparison between in-situ measured and calculated U-values and light transmittance values, and the structural assessment in relation to current requirements. These provide a basis for evaluating the reuse potential of existing glazing in façades and windows with metal framing.

### Typologies and dimensions of the investigated insulating glass units

This section provides an overview of the investigated IGUs and glass panes with respect to façade typologies, glazing assemblies, coating presence, and geometric characteristics.

Across the investigated buildings, the total registered installed IGU area amounted to approximately 18,300 m^2^, corresponding to an estimated glass mass of about 680 tones. In total, this corresponded to approximately 41,300 m^2^ of individual glass panes, of which about 21,100 m^2^ were uncoated and 20,200 m^2^ were coated. The large cumulative glazing area highlighted both the relevance of the investigated façades and their potential for reuse from a material and resource perspective.

**Fig. 7:**
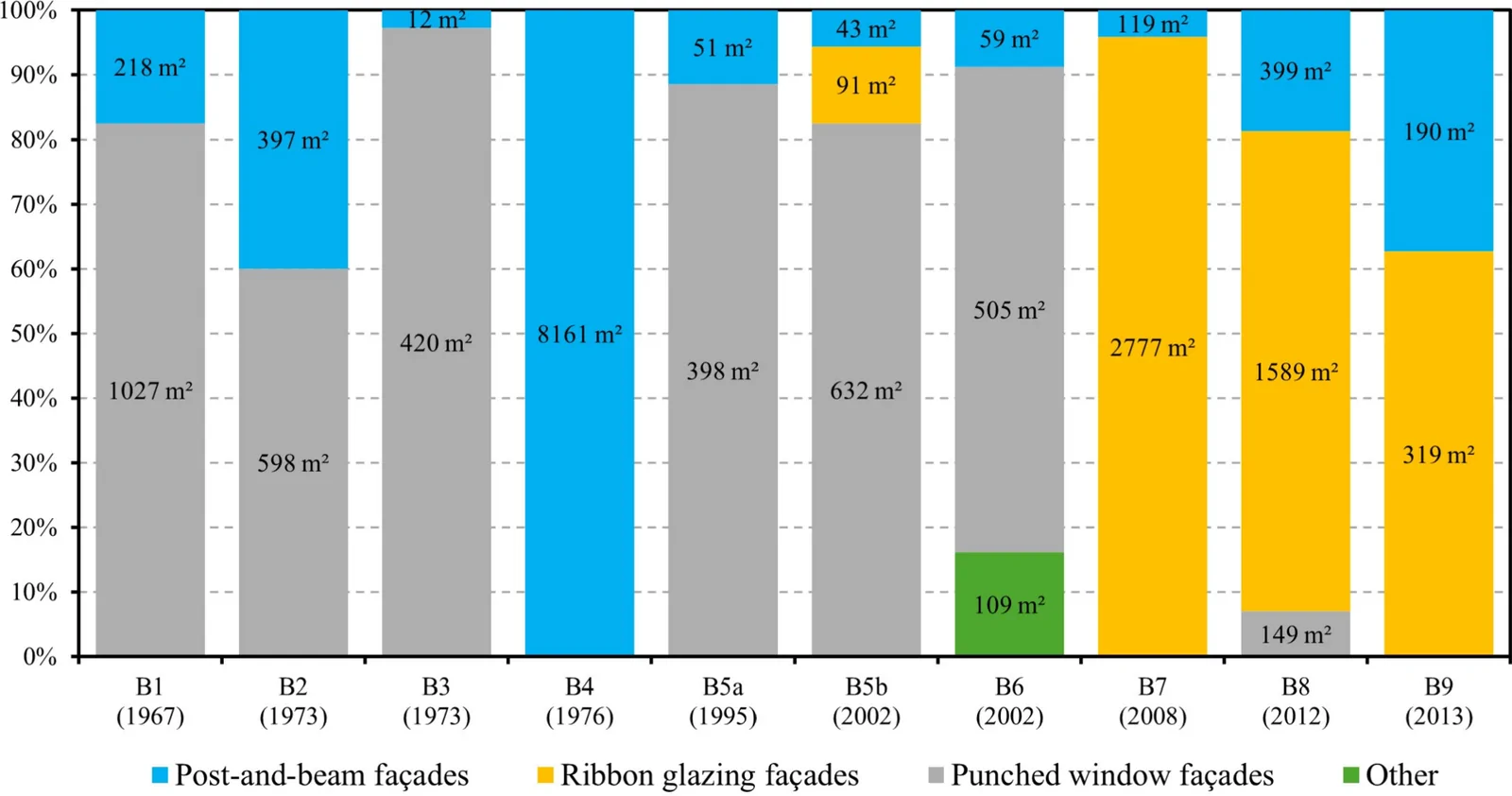
Distribution of the investigated IGUs by façade type and construction year, arranged in chronological order of the façade construction or refurbishment/replacement years

The distribution of the investigated IGUs by façade type and construction year is shown in Fig. 7. The collected data covered four main façade typologies, namely post-and-beam façades, ribbon glazing façades, punched window façades, and other façade systems. The buildings were arranged in chronological order according to the construction or renovation year of the façade, allowing the façade typologies to be directly related to the temporal development of the building stock. No system façades (unitized façades) were identified among the investigated buildings. It was further observed that several façades which appeared as ribbon glazing façades when viewed from the exterior were, in fact, executed as punched window façades, particularly in older buildings. In these cases, closely spaced window units were individually fixed to the building structure on all four sides. For the purpose of this study, such façades were categorized as punched window façades.

Post-and-beam façades were identified in all investigated buildings. This can be attributed to their frequent use at ground-floor levels and entrance areas. A further plausible explanation is that, unlike ribbon glazing and punched window façades, post-and-beam façades typically require IGUs to be replaced from the exterior in the event of damage. While this represents a practical advantage at ground-floor level, it becomes a disadvantage at upper floors, where replacement from the interior is generally preferable. Although these façades often accounted for a comparatively small share of the total glazed façade area, they were a recurring typology across the building stock.

Due to the limited number of buildings included in this study, a clear attribution of specific façade typologies to distinct construction periods could not be established. Nevertheless, the results indicated a general trend towards the replacement of punched window façades by façade systems that are less complex in terms of assembly and installation, suggesting a gradual shift towards more efficient façade solutions over time from the perspectives of installation speed and ease of construction. This can be explained by differences in the installation principles of the façade systems. Ribbon glazing façades are typically supported along the horizontal edges only, allowing higher levels of prefabrication, which significantly reduces installation time. In contrast, punched window façades typically consist of individually installed window units that are fixed on all four sides to the surrounding structure, making both installation and dismantling more time-consuming. A further relevant aspect for dismantling and refurbishment is the availability of detailed construction information. In the investigated buildings, detailed drawings of framing and connection details were often not available. In addition, as the buildings were in active use, it was not possible to remove claddings or other components to document these details.

The diagram also indicates the façade areas for the different façade types in each building. This shows that the different buildings significantly varied in size, which would allow for smaller or larger potential material stocks for eventual reuse projects.

**Fig. 8:**
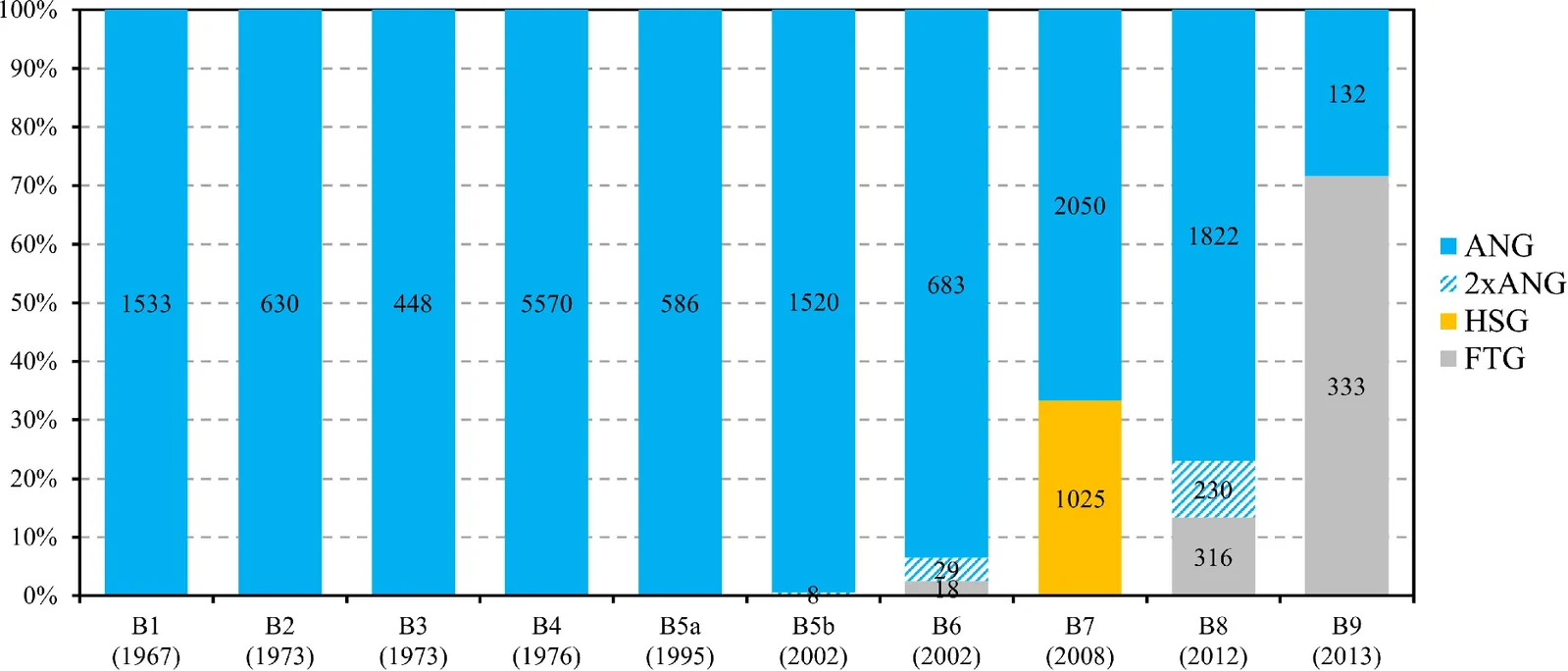
Distribution of glazing types of the investigated IGUs arranged in chronological order of the façade construction or refurbishment/replacement years, distinguishing ANG, HSG, FTG, and LSG

Figure 8 illustrates the distribution of glass types across the investigated buildings, arranged in chronological order of façade construction or refurbishment years, and differentiated by glass type, namely ANG, heat-strengthened glass (HSG), FTG, and LSG. In addition, the total number of glass panes (monolithic or laminated packages of IGUs) for each glass type is provided for each building.

Due to the limited number of buildings included in this study, a clear attribution of specific glass types to distinct construction periods could not be established. Nevertheless, the results indicated that older buildings predominantly featured ANG, whereas more recent buildings increasingly incorporated thermally toughened glass types, such as HSG and FTG. Despite this trend, ANG remained the most frequently used glass type across the investigated buildings.

The distribution of glass types is of particular relevance for reuse and remanufacturing strategies, as for thermally toughened glass it is considered that it cannot be cut or drilled, despite preliminary investigations in this direction by (Silvestru 2025). Generally, ANG allows for greater flexibility in further processing. Interestingly, laminated glass was only found in few of the investigated façades, although, according to current standards, several of the investigated IGUs would be required to fulfil barrier glazing requirements or would be classified as accessible. Consequently, the identified glass type distribution directly influences the technical feasibility and potential value of recovered glazing elements in the context of reuse and remanufacturing.

**Fig. 9:**
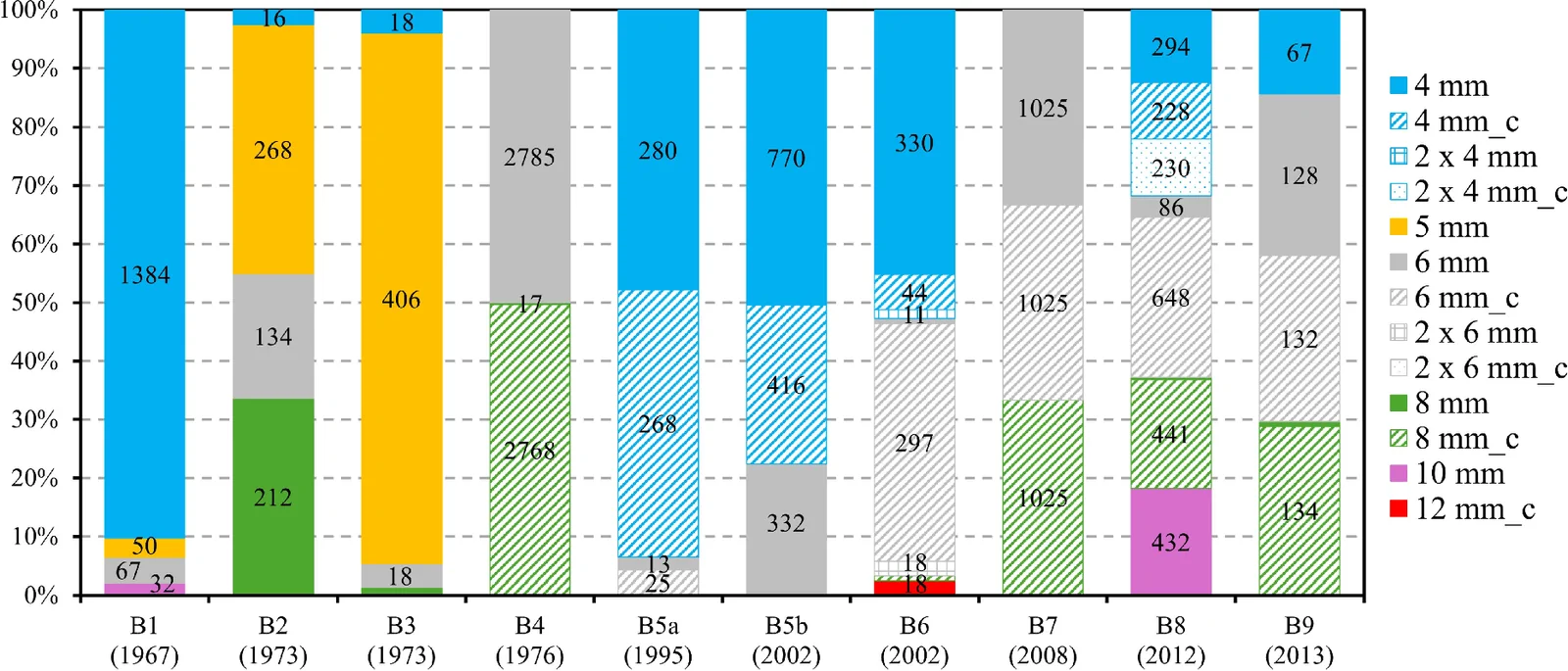
Distribution of glass thicknesses in the investigated IGUs arranged in chronological order of the façade construction or refurbishment/replacement years, and differentiated by the presence or absence of coatings. For clarity, labels of glass thicknesses occurring fewer than ten times were removed from the diagram

Figure 9 shows the distribution of glass thicknesses arranged in chronological order of the façade construction or refurbishment years, and differentiated by the presence or absence of coatings. In addition, the total number of glass panels (monolithic or laminated) with the respective thickness are provided for each building. Across the investigated buildings, glass thicknesses of 4 mm, 6 mm, and 8 mm were most frequently used, whereas glass panes with thicknesses of 10 mm or greater represented exceptions. The results further indicated that the outer glass panes were generally thicker than or equal in thickness to the inner panes, reflecting common design practice for insulating glass units.

With respect to coatings, the diagram shows that, for most buildings, either (*i*) all glass packages had no coatings, or if coatings were present, (*ii*) 50% of the glass packages were coated (for double IGUs), or (*iii*) 67% of the glass packages were coated (for triple IGUs). Minor deviations from these proportions could be attributed to entrance areas and other small façade regions. A more pronounced deviation was observed for building B5b, where the double IGUs facing office spaces were coated, while the double IGUs facing the stairwell were uncoated.

The information on the presence or absence of coatings is of particular importance, as coatings significantly influence the physical properties of glazing, including optical and thermal performance, and is therefore essential for assessing the feasibility and potential applications for glass reuse. Further details on the role of coatings and their impact on physical properties are provided in Sect. 3.2.

**Fig. 10:**
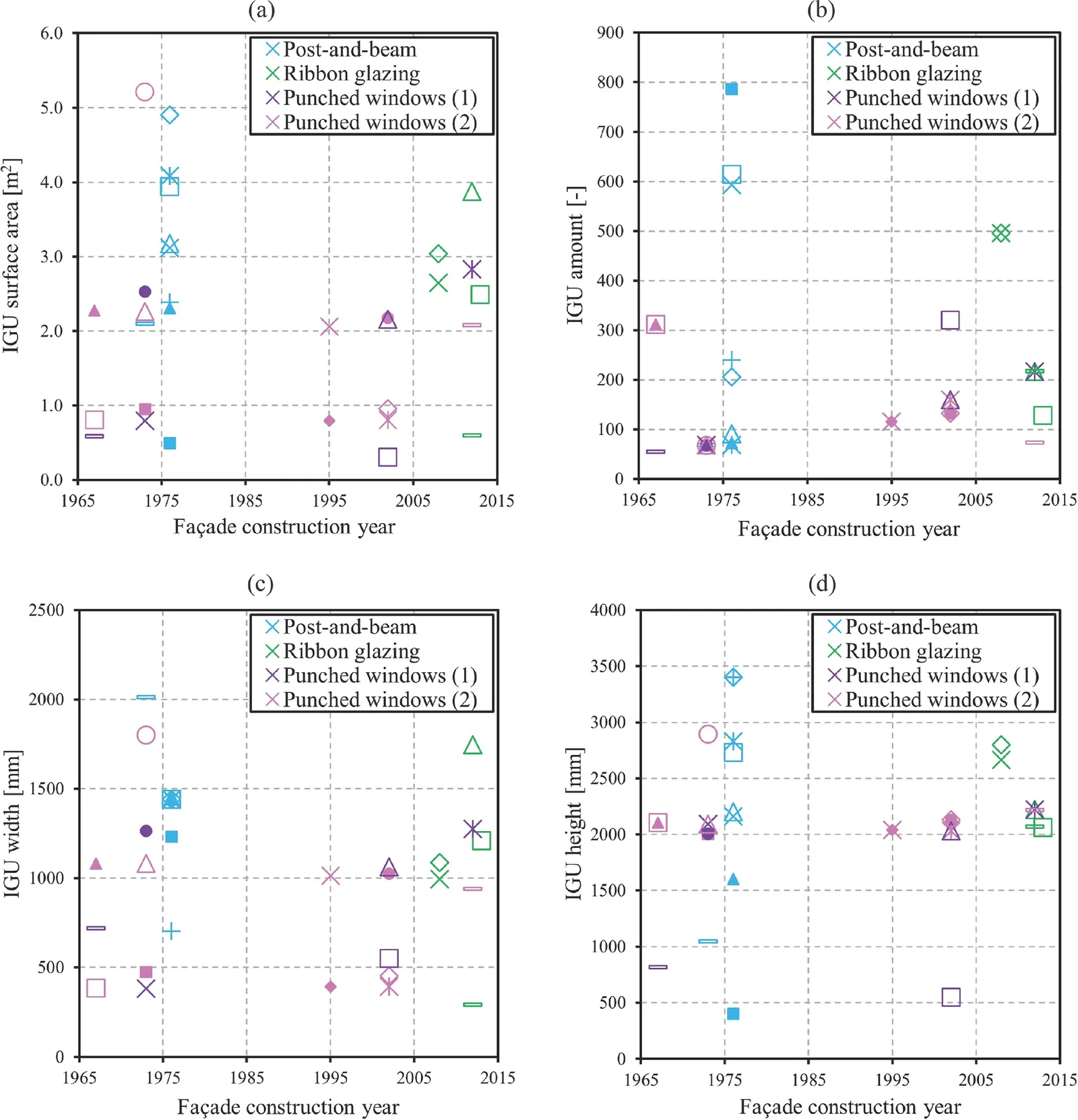
Geometric characteristics of the investigated IGUs, showing (**a**) IGU surface areas, (**b**) amounts, (**c**) widths, and (**d**) heights in relation to façade type. The symbols allow the corresponding entries in the figure to be linked across the different plots, while colors indicate the façade type. For punched window façades, two different colors are used to improve the readability of the figure

The geometric characteristics of the investigated IGUs are summarized in Fig. 10, which relates IGU surface areas (Fig. 10a), IGU amounts (count numbers; Fig. 10b), IGU widths (Fig. 10c), and IGU heights (Fig. 10d) to the different façade types. The figure illustrates the substantial variability in glazing geometry across façade systems.

**Fig. 11:**
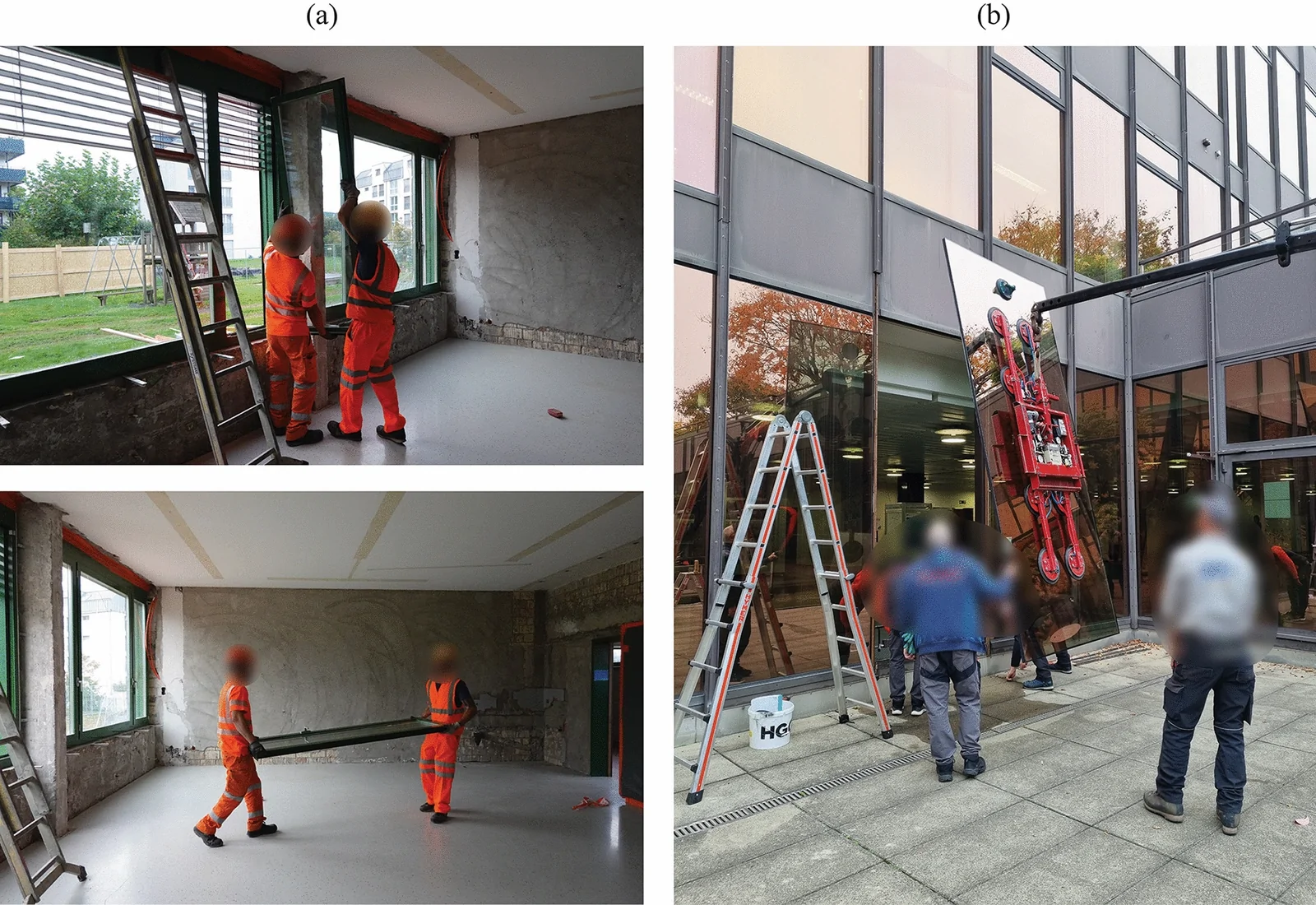
Influence of IGU size on dismantling feasibility: (**a**) example of relatively small IGUs in window sashes that can be dismantled and handled manually, and (**b**) large IGUs in post-and-beam façades whose weight prevents manual dismantling and requires mechanical assistance

The geometric information, in combination with the amounts of identical IGUs, is of particular relevance for reuse and remanufacturing scenarios. In general, higher quantities of identical glazing units can facilitate more efficient dismantling processes, as equipment and setup efforts can be distributed across a larger number of units and learning effects may progressively accelerate the dismantling process. The influence of IGU size on reuse and remanufacturing potential is less straightforward. Larger IGUs offer increased flexibility for cutting and remanufacturing into new dimensions, potentially enabling a wider range of reuse and remanufacturing applications. At the same time, however, increased glass weight significantly complicates dismantling, as larger IGUs become more difficult to handle and may no longer be safely transportable by hand. In addition, the risk of failure of the aged edge seal increases during dismantling of larger IGUs, as the higher self-weight may induce additional shear stresses on the sealing system during handling and transport. In contrast, smaller IGUs can typically be dismantled manually, facilitating handling and reducing the risk of damage during removal. To illustrate these aspects, Fig. 11 shows two examples—one for a punched window façade with medium-size window sashes that could still be removed and transported by hand (Fig. 11a), and one for a post-and-beam façade with large insulating glass units weighting around 200 kg, for which special mechanized tools were necessary to remove and transport the glazing unit (Fig. 11b).

### Comparison between in-situ-measured and calculated U-values and light transmission values

This section compares the in-situ measured U-values and light transmission values with software-based calculated values for the investigated IGUs. The results are summarized in Table 2, which provides an overview for each façade, including the glazing assembly, the presence and position of coatings, and the estimated installation year of the glass. The table allows a direct comparison between measured and calculated optical and thermal properties for the investigated glazing assemblies.

**Fig. 12:**
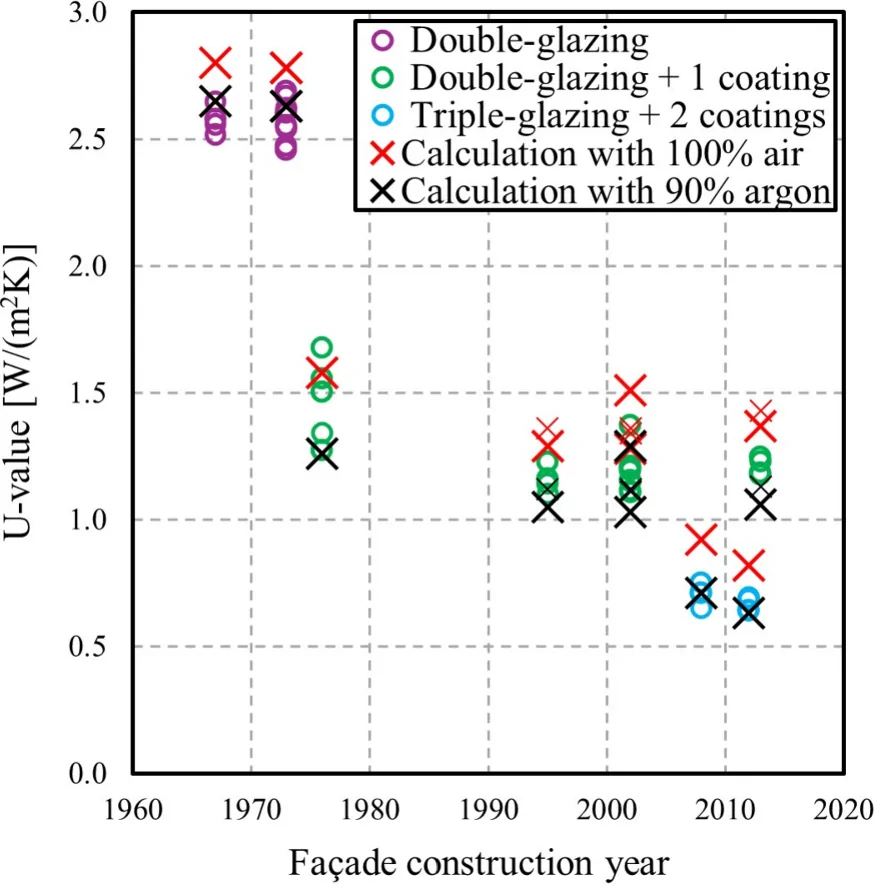
Comparison between in-situ measured and calculated U-values of the investigated IGUs. Measured U-values are shown color-coded according to the glazing assembly. Calculated U-values assuming 100% air-filled cavities and 90% argon-filled cavities are included for comparison. Additional smaller markers indicate calculated U-values using the standard Glas Trösch coating EN2plus

**Table 2:** Overview of façade-specific glazing assemblies including coating position and estimated installation year of the glass, together with measured and calculated optical (UV, VL) and thermal (U-value) performance. Measured U-values are reported with standard deviation and calculated U-values are reported for air-filled (100%) and argon-filled (90%) cavity assumptions, respectively. For the values indexed with “1”, the bracketed values in the second row correspond to calculations performed using the standard low-E coating EN2plus

Building	Glazing assembly	Coating position	Inst. year*	UV_meas_ (%)	VL_meas_ (%)	U_meas_ ± SD (W/m^2^K)	UV_calc,1_ (%)	VL_calc,1_ (%)	U_calc,1_ (W/m^2^K)	UV_calc,2_ (%)	VL_calc,2_ (%)	U_calc,2_ (W/m^2^K)
B1	4 / 12 / 4	None	1967	66	83	2.57 ± 0.05	56	83	2.80 | 2.65	55	82	2.9 | 2.7
B2	5 / 12 / 5	None	1973	60	83	2.54 ± 0.09	53	82	2.78 | 2.63	51	81	2.8 | 2.7
B3	5 / 12 / 5	None	1973	63	85	2.61 ± 0.08	53	82	2.78 | 2.63	51	81	2.8 | 2.7
B4	8 / 12 / 6	2	1976**	13	26	1.47 ± 0.16	13^a^ (29)	27^a^ (81)	1.58 | 1.26^a^ (1.58 | 1.26)	11^f^	24^f^	1.5 | 1.2^f^
B5a	4 / 18 / 4	3	1995	22	75	1.16 ± 0.05	25^b^ (33)	73^b^ (82)	1.29 | 1.05^b^ (1.36 | 1.12)	25^g^	77^g^	1.3 | 1.1^g^
B5b	6 / 18 / 4	3	2002	38	76	1.23 ± 0.09	33^c^ (32)	81^c^ (81)	1.51 | 1.29^c^ (1.36 | 1.12)	42^h^	81^h^	1.4 | 1.1^h^
B6	6 / 16 / 4	2	2002	5	69	1.14 ± 0.04	5^d^ (32)	62^d^ (81)	1.28 | 1.03^d^ (1.34 | 1.11)	9^i^	70^i^	1.3 | 1.0^i^
B7	8 / 12 / 6 / 12 / 6	2,5	2008	NM	NM	0.71 ± 0.04	–	–	0.92 | 0.71^e^	–	–	0.9 | 0.7^h^
B8	8 / 14 / 10 / 14 / 6	2,5	2012	NM	NM	0.67 ± 0.03	–	–	0.82 | 0.63^e^	–	–	0.8 | 0.6^h^
B9	6 / 14 / 6	2	2013	6	65	1.21 ± 0.03	5^d^ (30)	61^d^ (81)	1.37 | 1.06^d^ (1.43 | 1.13)	9^i^	69^i^	1.4 | 1.1^i^

^*^The reported installation year generally corresponds to the façade construction or refurbishment year and is typically 1–2 years later than the production date indicated on the IGU spacer

^**^For Building B4, a larger number of IGUs located in the upper floors were identified with spacer production dates from 1979, approximately three years later than the official façade completion year. These glazing units exhibited the same glass assembly configuration but revealed comparatively low U-values during the measurements, indicating the possible presence of noble gas filling (e.g., argon) within the cavities. This aspect should be investigated in future studies

^a^Chosen coating: COMBI Neutral 30 T, ^b^chosen coating: SELEKT 74/42 T, ^c^chosen coating: TRIII E, ^d^Chosen coating: COMBI Neutral 61/32, ^e^chosen coating: EN2plus, ^f^chosen coating: Stopray Silver 25/17 ^g^chosen coating: iplus 1.0, ^h^chosen coating: iplus 1.1, ^i^chosen coating: Energy 70/37

For each investigated IGU configuration, the UV transmission and VL transmission were measured on multiple glazing units and reported as consolidated representative values. The measured U-values were evaluated statistically for the four measurements per glazing configuration and are presented as mean values with corresponding standard deviations (U_meas_ ± SD), reflecting the variability between the individual in-situ measurements.

In addition, software-based calculated values for UV transmission, VL transmission, and U-value are reported for each glazing configuration. The calculated results for the U-value are provided for two boundary conditions, assuming 100% air-filled cavities (first value) and 90% argon-filled cavities (second value), allowing a direct comparison with the measured performance. Since gas content measurements were not conducted, the original gas filling of the investigated IGUs could not be determined. Therefore, the objective of the comparative calculations was not to identify the exact gas composition, but to assess whether the measured U-values fall within the range defined by simplified assumptions based on the measured glass assembly and the identified presence or absence of coatings. Values indexed with “1” were calculated using the software SILVERSTAR glaCE (Glas Trösch), whereas values indexed with “2” were obtained using the Glass Configurator (AGC Glass Europe). For the values indexed with “1”, the bracketed values in the second row correspond to calculations performed using the standard low-emissivity (low-E) coating EN2plus.

Although (DIN EN 673, 2025) notes that reporting U-values with more than one decimal place may convey a misleading impression of accuracy, U-values in this study are reported with two decimal places to enable a better differentiation of the measured values. This level of precision was consistently applied to the measured data and to the results obtained from SILVERSTAR glaCE. The results derived from the Glass Configurator are presented with the precision provided by the software, as the output format does not allow for adjustment of the number of decimal places. Despite minor numerical differences, the calculated values show a high level of agreement, indicating consistent modelling assumptions across both tools.

The comparison of U-values is illustrated in Fig. 12, which shows the measured U-values of all investigated IGUs, color-coded according to the glazing assembly, together with the calculated U-values assuming 100% air filling and 90% argon filling. For clarity, the calculated values are displayed only once per IGU, as the results obtained from both software tools are nearly identical. In addition to the calculations based on the coatings selected to best match the measured optical properties, Fig. 12 also includes results calculated using the most commonly applied standard coating of Glas Trösch (EN2plus). These results are shown as smaller markers in the same color as the corresponding glazing assemblies and serve as a reference for comparing the influence of coating selection on the calculated U-values.

It should be noted that the comparison between measured and calculated U-values does not represent a validation of the measurement results or the applied calculation approach. The objective of the calculations was rather to assess whether simplified and practically applicable assumptions can provide reasonable estimates of the thermal performance of existing IGUs. Therefore, the comparison should be interpreted as an indicative assessment of simplified estimation methods rather than a strict validation of measured or calculated values.

The diagram indicates that the choice of coating has only a minor influence on the calculated U-values of the investigated IGUs. This suggests that, for the purpose of estimating the U-value of aged glazing, the use of a standard low-emissivity coating in combination with a conservative assumption of 100% air-filled cavities provides, in general, a reliable and safe approximation. Under these assumptions, the time-consuming in-situ measurement of U-values may be avoided, while still obtaining representative estimates of the thermal performance of existing IGUs. Typical U-values for modern IGUs are in the range of approximately 1.0–1.1 W/m^2^K for double glazing and around 0.6—0.7 W/m^2^K for triple glazing, depending on the specific configuration, gas filling, and coating type (AGC Glass Europe; Glas Trösch). The investigated IGUs generally exhibit higher U-values, reflecting the standards at the time of installation. However, the measured values still indicate a performance level that may be sufficient for certain reuse applications, depending on the specific requirements, or that could be upgraded to a comparable level through remanufacturing.

When comparing U-values reconstructed from the measured R-values using standardized internal and external surface resistances (R_si_ and R_se_) with directly measured U-values, deviations of up to approximately 10% were observed. These differences arise because the directly measured U-values implicitly include actual surface resistances, which depend on the specific environmental conditions during the measurement period (e.g., wind conditions and installation conditions). In contrast, the reconstructed U-values are based on standardized surface resistances, enabling a consistent comparison with calculated values. Therefore, while the directly measured U-values accurately reflect the in-situ conditions during the measurement, they are not directly comparable to calculated values based on standardized conditions. The comparison should therefore be interpreted as an indicative consistency check rather than a validation.

Almost all measured U-values were located between the U-values obtained from the two calculation approaches. However, all measured U-values except one indicated a better thermal performance than the calculated values assuming exclusively air-filled cavities.

**Fig. 13:**
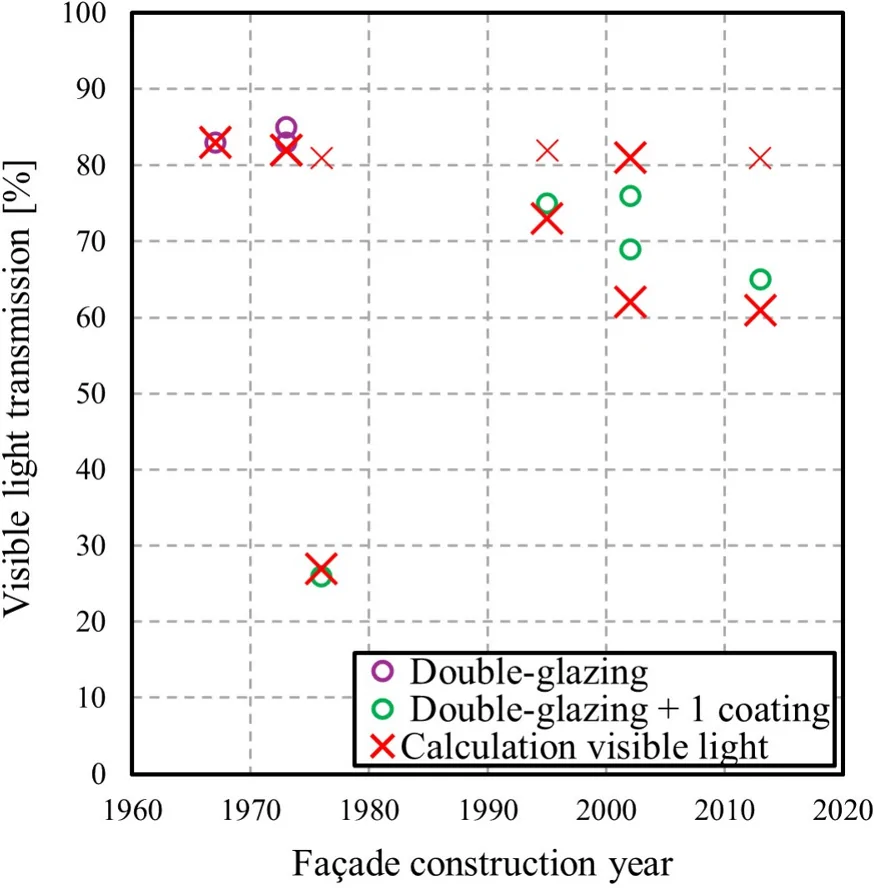
Comparison between in-situ measured and calculated visible light transmission (VL) of the investigated IGUs. Measured visible light transmissions are shown color-coded according to the glazing assembly. Calculated visible light transmissions are included for comparison. Additional smaller markers indicate calculated visible light transmissions using the standard Glas Trösch coating EN2plus

**Fig. 14:**
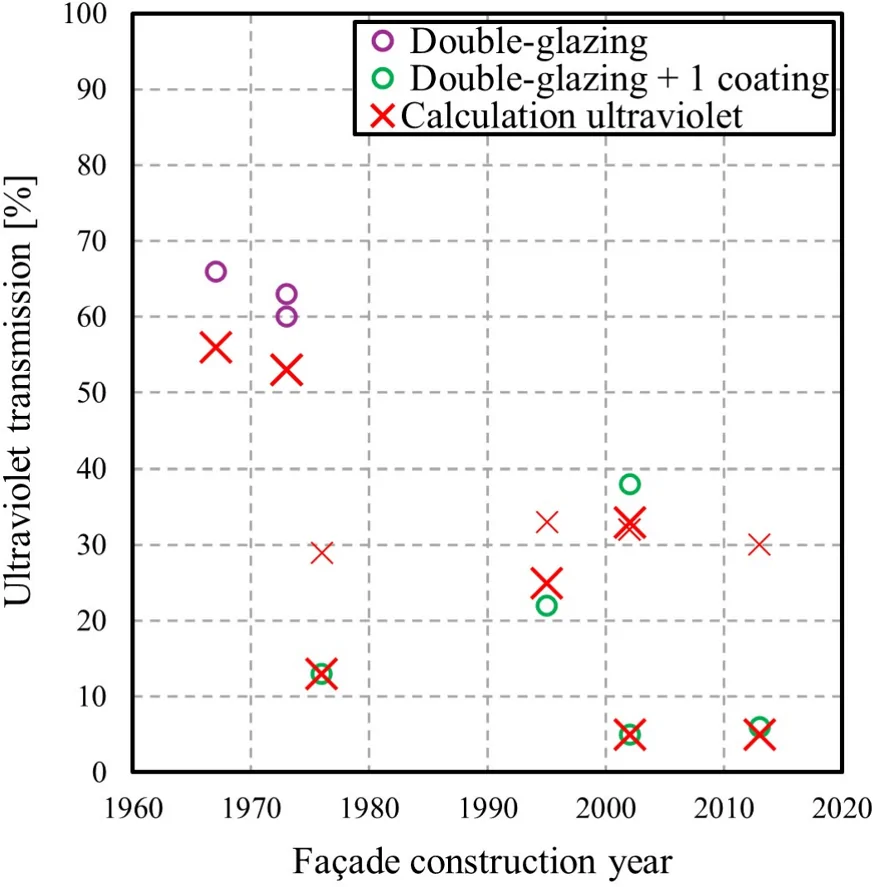
Comparison between in-situ measured and calculated ultraviolet transmission (UV) of the investigated IGUs. Measured ultraviolet transmissions are shown color-coded according to the glazing assembly. Calculated ultraviolet transmissions are included for comparison. Additional smaller markers indicate calculated ultraviolet transmissions using the standard Glas Trösch coating EN2plus

In addition to the comparison of U-values, the evaluation was extended to the optical performance of the investigated IGUs. Figure 13 illustrates the comparison between measured and calculated VL transmission and Fig. 14 shows the comparison between measured and calculated UV transmission. Analogous to the U-value comparison, the diagrams include both in-situ measured values and software-based calculated results. In addition to the calculations based on the coatings selected to best match the measured optical properties, Figs. 12, 13 and 14 also includes results calculated using the most commonly applied standard low-E coating of Glas Trösch (EN2plus). These results are shown as smaller markers (x) in the same color as the corresponding glazing assemblies and serve as a reference for comparing the influence of coating selection on the calculated VL and UV transmission values. This approach allowed a direct comparison between measured and calculated optical properties and provides additional insights into the suitability of using standard coating assumptions when estimating the performance of existing and aged IGUs. Light transmission measurements could not be performed for the triple-glazed IGUs, since the available measurement device, which was U-shaped and needed to be positioned over the glazing unit edge could not be applied. This was either because the window or façade elements were not openable, or because the framing was thicker than the U-shaped measurement device. Consequently, no measured VL or UV transmission values for triple glazing are included in the diagrams.

The high deviation between the measured VL transmission and the corresponding calculated value in Fig. 13 for one of the façades was due to a highly reflective solar coating (see building B4 in Fig. 2), whose properties deviate strongly from those of the standard coating used in the calculations.

In contrast to the U-value comparison, the results for VL and UV transmission revealed that the choice of coating type from those available from current producers can significantly influence a prediction for the properties of an aged insulating glass unit. The diagrams indicate that assuming one of today’s standard low-emissivity coatings in the calculations would generally lead to higher VL and UV transmission values compared to those achieved by glazing units with older coatings. This is reflecting the ongoing optimization of optical performance in coated glazing systems; however, it should be considered that in general it is not known what coatings were available when the investigated façades were designed, and what the selection criteria was for the coatings.

Modern IGUs with low-E coating typically achieve visible light transmission values in the range of approximately 70–80%, depending on the glazing configuration and coating type (AGC Glass Europe; Glas Trösch). The investigated IGUs generally exhibited lower visible light transmission values, reflecting the coating technologies and performance requirements at the time of installation. Nevertheless, the measured optical properties may still be suitable for certain reuse applications, depending on the daylighting and functional requirements of the intended application, or could potentially be improved through remanufacturing strategies.

For these reasons, light transmission properties should be measured and evaluated prior to any reuse or remanufacturing application of coated glass, in order to ensure that the optical performance of the glazing meets the requirements of the intended new use.

### Structural assessment in relation to current requirements

This section presents the results of the structural assessment of the investigated IGUs. An overview of the evaluated glazing units is provided in Table 3, which lists the building number, glazing assembly, IGU width, IGU height, and the corresponding maximum admissible wind load (wind pressure and wind suction) for each IGU (the higher absolute value between wind pressure and wind suction is provided). In addition, the characteristic stresses in the glazing under the relevant design load combination (wind load and climatic load) of the representative reference building are reported. Next to the maximum admissible wind loads, the governing design check situation is provided in brackets (ultimate limit state or serviceability limit state). A clear dependency of the governing design check on the width of the glazing could not be identified, since also the glass thicknesses and the glass type play a significant role. Identifying such dependencies and limits would be of interest in the context of reuse. If the serviceability limit state would be mostly governing starting with certain dimensions of the glazing units, a reduction of glass strength due to ageing effects would be of significantly lower interest. The investigations in this regard will be continued after a larger database of glazing assemblies and sizes will be collected.

**Table 3:** Overview of façade-specific glazing assemblies with IGU dimensions and corresponding maximum admissible characteristic wind loads, and characteristic stresses under the relevant design load combination (wind load and climatic load) of the representative reference building, derived from the structural assessment

Building	Glazing assembly	Width (mm)	Heigh (mm)	Maximum admissible wind load (kN/m^2^)	Characteristic stress (N/mm^2^)
B1	4 ANG / 12 / 4 ANG	1082	2103	1.20 (SLS)	28.0^a^
B1	4 ANG / 12 / 4 ANG	383	2103	1.10*	–
B1	4 ANG / 12 / 4 ANG	715	812	> 3.00 (ULS)	23.1^b^
B2	8 ANG / 12 / 8 ANG	2010	1041	> 3.00 (ULS)	18.7^a^
B2	5 ANG / 12 / 5 ANG	1263	2004	2.10 (SLS)	23.1^a^
B2	5 ANG / 12 / 5 ANG	473	2004	1.10*	–
B2	6 ANG / 12 / 6 ANG	1800	2895	1.50 (SLS)	27.1^a^
B3	5 ANG / 12 / 5 ANG	1082	2091	2.00 (SLS)	27.4^a^
B3	5 ANG / 12 / 5 ANG	380	2091	1.10*	–
B4	8 ANG / 12 / 6 ANG	1442	2730	2.40 (ULS)	27.7^a^
B4	8 ANG / 12 / 6 ANG	1442	3400	1.70 (ULS)	32.6^a^
B4	8 ANG / 12 / 6 ANG	1442	2200	> 3.00 (ULS)	23.6^a^
B4	8 ANG / 12 / 6 ANG	1442	2160	> 3.00 (ULS)	23.1^a^
B4	8 ANG / 12 / 6 ANG	1442	2830	2.30 (ULS)	28.6^a^
B4	8 ANG / 12 / 6 ANG	1442	1600	> 3.00 (ULS)	18.4^a^
B4	8 ANG / 12 / 6 ANG	1230	400	1.10*	–
B4	8 ANG / 12 / 6 ANG	702	3400	> 3.00 (ULS)	34.6^b^
B5a	4 ANG / 18 / 4 ANG	1012	2038	1.20 (SLS)	30.4^a^
B5a	4 ANG / 18 / 4 ANG	391	2038	0	70.6^b^
B5b	4 ANG / 16 / 4 ANG	550	550	1.10*	–
B5b	6 ANG / 18 / 4 ANG	1063	2030	2.10 (SLS)	30.3^a^
B5b	6 ANG / 18 / 4 ANG	393	2030	1.10*	–
B6	6 ANG / 16 / 4 ANG	1023	2129	1.90 (ULS)	31.0^a^
B6	6 ANG / 16 / 4 ANG	451	2129	1.10*	–
B7	8 ANG / 12 / 6 HSG / 12 / 6 ANG	1086	2800	2.50 (ULS)	39.1^b^
B8	8 ANG / 14 / 10 ANG / 14 / 6 ANG	1745	2220	> 3.00 (ULS)	18.6^b^
B8	8 ANG / 14 / 10 ANG / 14 / 6 ANG	1274	2220	> 3.00 (ULS)	34.0^b^
B8	4 .4.2 ANG / 14 / 4 ANG / 14 / 4 ANG	287	2066	1.10*	–
B8	6 FTG / 14 / 4 FTG / 14 / 6 FTG	937	2212	> 3.00 (SLS)	No ANG
B9	6 FTG / 16 / 6 ANG	1208	2061	2.90 (SLS)	24.4^a^

^*^The simplified verification according to Clause 5.4.3.8 of (SIA 2057 2021)

^a^Indicates the relevant design load combination with wind load governing

^b^Indicates the relevant design load combination with climatic load governing (without wind)

For the selected IGUs, the maximum admissible wind loads were determined for the measured glass assemblies. In the structural calculations, the maximum admissible characteristic wind loads were capped at 3.00 kN/m^2^. This upper limit was introduced not only because wind actions exceeding this magnitude were expected to occur only rarely within the Zurich region, but also to improve the clarity and comparability of the graphical and tabulated presentation of the results. Consequently, IGUs reaching this upper limit may, in reality, be capable of resisting significantly higher wind loads. However, the applied cap did not affect the interpretation of structural suitability for the typical building applications considered in this study.

**Fig. 15:**
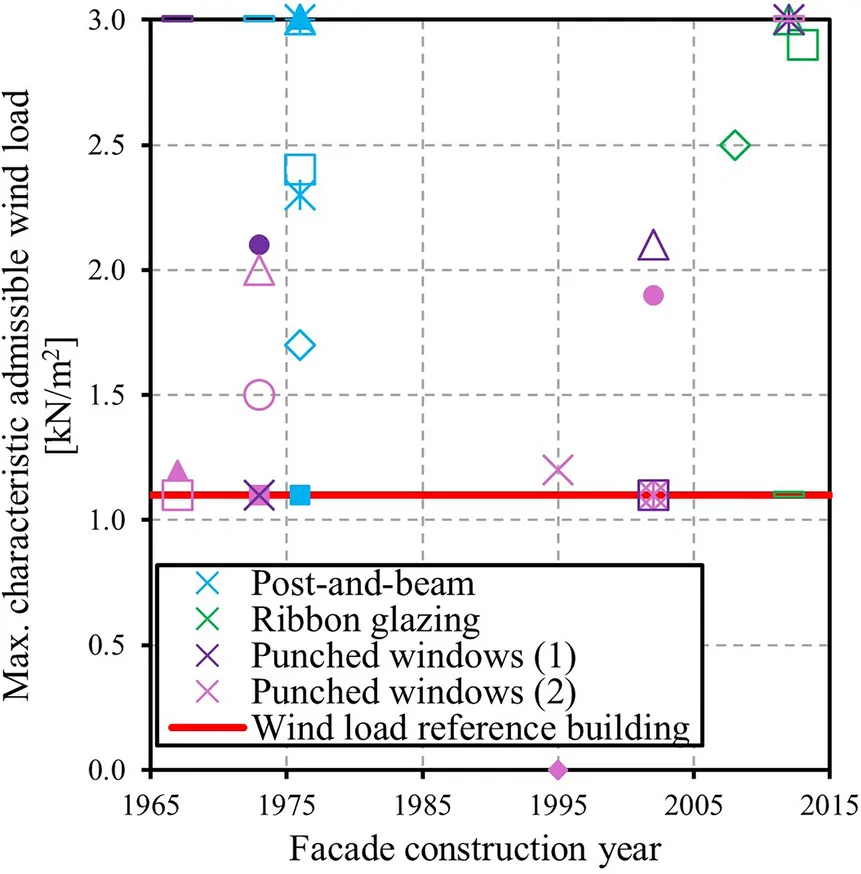
Maximum admissible characteristic wind loads of the investigated IGUs. The horizontal red line indicates the characteristic wind load of the representative reference building (w_k_ = 1.10 kN/m^2^) described in Sect. 2.5. IGUs with admissible wind loads above this line may be considered structurally suitable for installation in the reference building under the assumed boundary conditions. Values are capped at 3.0 kN/m^2^ for better readability of the diagram

The distribution of the maximum admissible wind loads of the investigated IGUs is illustrated in Fig. 15, where the characteristic wind load w_k_ = 1.10 kN/m^2^ is indicated by a horizontal red reference line. All IGUs with admissible wind loads exceeding this value are located above the red line and may therefore be considered structurally suitable for installation in the representative building described in Sect. 2.5, as they would be capable of resisting wind loads higher than those required under the assumed boundary conditions.

One result yielded a maximum admissible wind load of zero, for which the simplified verification according to Clause 5.4.3.8 could not be applied, as the insulating glass unit exceeded the maximum allowable cavity width with a cavity spacing of 18 mm. In this case, additional project-specific investigations would be required, particularly with respect to the actual local height difference ΔH, in order to establish a reliable structural verification. Such assessments were beyond the scope of this study and would need to be addressed in case of a reuse project.

Except for one glazing configuration, all investigated IGUs would be permissible for installation in the assumed reference building, provided that the glazing is classified as non-accessible and without a barrier glazing function. While a definitive statement on the proportion of existing glazing that complies with current structural requirements could not be established due to the limited number of buildings, the results nevertheless indicate that a large share of the investigated IGUs would likely still satisfy today’s structural criteria under comparable boundary conditions.

**Fig. 16:**
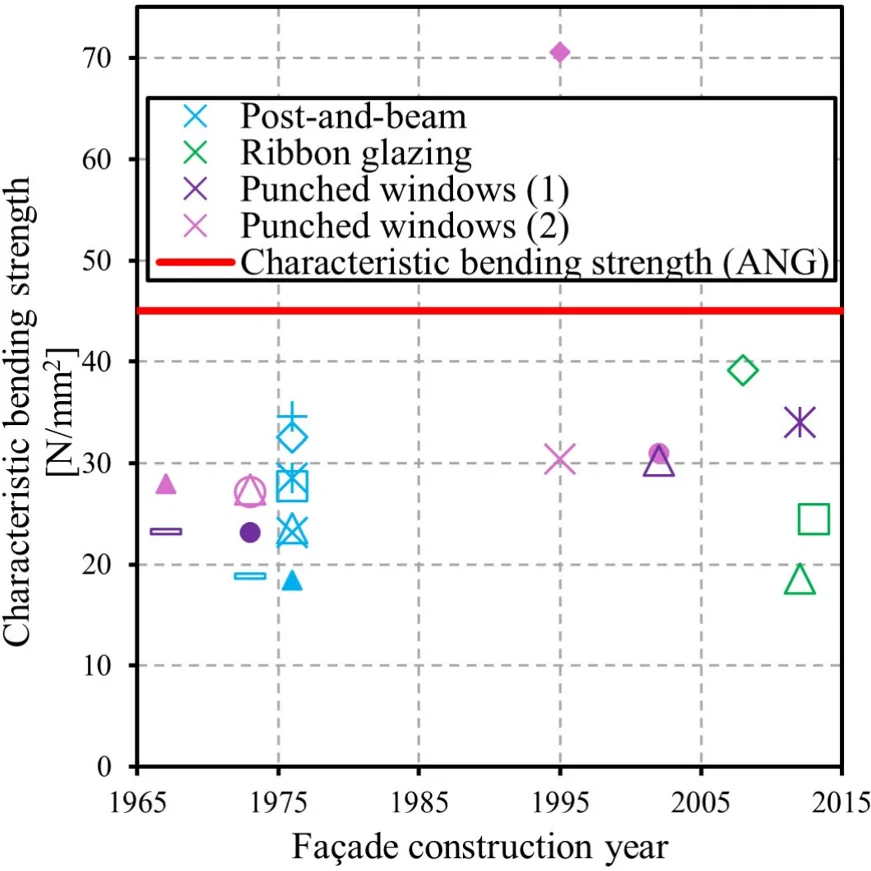
Characteristic stresses in the investigated IGUs under the relevant design load combination (wind load and climatic load) of the representative reference building compared to the characteristic bending strength of annealed glass (45 N/mm^2^). Values exceeding the strength limit indicate insufficient structural capacity under the assumed boundary conditions

In addition to the wind load-based assessment, Fig. 16 presents the characteristic stresses expected in the glazing under the relevant design load combination (wind load and climatic load) of the representative reference building in comparison with the characteristic bending strength of annealed glass. This comparison provides a more differentiated assessment of the structural performance and allows an estimation of the minimum characteristic glass strength required for the given glazing geometries, considering that this strength might be lower for aged glass compared to new one.

The results indicate that, for most glazing units, the stress levels remain below the characteristic bending strength assumed for new annealed glass, suggesting that these units could remain structurally sufficient even if a certain level of strength degradation is considered. However, one glazing configuration exceeds the characteristic strength limit of 45 N/mm^2^. This result is consistent with the previously identified non-verifiable case in Fig. 15 and reflects the same governing critical configuration.

## Conclusions

This study investigated the condition and reuse potential of insulating glass units (IGUs) from existing façades and windows by combining documentation analysis, in-situ measurements, software-based performance calculations, and structural verification according to current standards. Nine buildings with metal-framed façades constructed or refurbished between 1967 and 2013 were analyzed, providing a representative overview of glazing typologies, assemblies, dimensions, and performance levels found in the investigated building stock.

The results show that a substantial amount of glazing is available for potential reuse, amounting to approximately 18,300 m^2^ of IGUs or about 680 tons of glass. The investigated façades exhibit a wide range of glazing geometries and façade typologies, with punched window façades, ribbon glazing façades, and post-and-beam façades all being present. While a clear attribution of façade typologies to specific construction periods could not be established due to the limited number of buildings, a trend towards less assembly-intensive façade systems was observed. The geometric characteristics and quantities of identical IGUs were identified as key parameters for reuse and remanufacturing, as higher repetition facilitates dismantling efficiency, whereas large IGU dimensions increase handling complexity and the risk of edge seal failure.

The comparison between in-situ measured and calculated U-values indicated that most measured values lay between the calculated results assuming 100% air-filled and 90% argon-filled cavities. Measured U-values were consistently better than calculations assuming exclusively air-filled cavities. Furthermore, the influence of coating selection on U-values was found to be minor. This suggests that, for the assessment of aged glazing, the use of a standard low-emissivity coating combined with a conservative assumption of air-filled cavities provides a robust and practical approximation, potentially reducing the need for time-consuming in-situ U-value measurements.

In contrast, the evaluation of visible light (VL) and ultraviolet (UV) transmission revealed a clearer differentiation between glazing generations. Glazing units with more recent coatings exhibited higher VL and UV transmission compared to such with older coatings, highlighting the importance of optical performance assessment prior to considering reuse or remanufacturing. Consequently, light transmission properties should be measured and evaluated before each intended reuse application to ensure compatibility with current daylighting and functional requirements.

The structural assessment demonstrated that, with one exception, all investigated IGUs would be permissible for installation in a representative reference building from Zurich under the assumption of non-accessible glazing. This finding indicates that a large share of existing IGUs may still comply with current structural requirements in terms of load-bearing capacity and meeting deflection limits (ultimate limit state and serviceability limit state). However, because of the used glass types, they would often fail to meet current standards in terms of safety and residual load-bearing capacity (fracture limit state and post-fracture limit state). Nevertheless, a more comprehensive assessment involving a larger number of buildings would be required to draw statistically robust conclusions. Moreover, strength reduction of glass because of ageing effects, and long-term behavior of IGUs, particularly with respect to gas pressure equalization and edge seal durability, remain important aspects for future research.

The study further highlighted the considerable effort required to acquire reliable geometric data for existing façades through the evaluation of archival documents and detailed field investigations. In this context, future research could explore the development of automated preliminary analysis methods, for example by combining machine learning–based image analysis with geographic information system (GIS) data, to support large-scale identification of relevant buildings and quantification of reusable façade elements.

Overall, the results demonstrated that a significant proportion of existing IGUs has the potential to be reused under current performance and structural criteria, or even better, that their current use is extended. The study highlighted key parameters influencing reuse feasibility, including glazing geometry, repetition, coating characteristics, and accessibility. These findings provide a valuable basis for developing future circular façade strategies and underline the importance of systematic condition assessment methods when considering the reuse and remanufacturing of glazing from the existing building stock.

## Data Availability

The data required to produce these findings are part of a running research project. The data will be made available on request sent to the corresponding authors, either directly or after completion of the research project.

## References

[CR1] AGC Glass Europe Thermobel—One brand name for all AGC insulating double or triple glazing units. Retrieved 28 Apr 2026 from https://www.agc-yourglass.com/en-BE/brands/thermobel#productListRangeBlock

[CR2] Allwood, J.M., Ashby, M.F., Gutowski, T.G., Worrell, E.: Material efficiency: a white paper. Resour. Conserv. Recycl. **55**(3), 362–381 (2011). 10.1016/j.resconrec.2010.11.002

[CR3] Augiseau, V., Barles, S.: Studying construction materials flows and stock: a review. Resour. Conserv. Recycl. **123**, 153–164 (2017). 10.1016/j.resconrec.2016.09.002

[CR4] Çetin, S., Raghu, D., Honic, M., Straub, A., Gruis, V.: Data requirements and availabilities for material passports: a digitally enabled framework for improving the circularity of existing buildings. Sustain. Prod. Consum. **40**, 422–437 (2023). 10.1016/j.spc.2023.07.011

[CR5] Cupać, J., Datsiou, K.C., Louter, C.: Reuse potential of architectural glass: experimental study on the strength of used window glazing. Glass Struct. Eng. **9**(3), 321–337 (2024). 10.1007/s40940-024-00267-y

[CR6] Davis, C., Granvik, T., Yin, K., Sellman, R., Back, R., Scherer, C., Giovanni, L.: Sustainable Glass Facades: Understanding the Long-Term Thermal Performance of IGUs. In: GPD (eds.) GPD Glass Performance Days 2025. Tampere, Finland (2025)

[CR7] DeBrincat, G., Babic, E.: Re-thinking the life-cycle of architectural glass. Construction flat glass recycling - Viability study & value report. Arup (2018). Retrieved 12 Mar 2025 from https://www.arup.com/globalassets/downloads/insights/re-thinking-the-life-cycle-of-archtectural-glass.pdf

[CR8] DIN 18008-2. Glass in Building—Design and construction rules—Part 2: Linearly supported glazings (2020)

[CR9] DIN EN 673. Glass in building—Determination of thermal transmittance (U value)—Calculation method (2025)

[CR10] European Commission. A Renovation Wave for Europe—greening our buildings, creating jobs, improving lives (2020). https://eur-lex.europa.eu/legal-content/EN/TXT/?uri=CELEX:52020DC0662

[CR11] Feng, Y., Duan, Q., Wang, J., Baur, S.: Approximation of building window properties using in situ measurements. Build. Environ. **169**, 106590 (2020). 10.1016/j.buildenv.2019.106590

[CR12] Geboes, E., Galle, W., De Temmerman, N.: Make or break the loop: a cross-practitioners review of glass circularity. Glass Struct. Eng. **8**(2), 193–210 (2023). 10.1007/s40940-022-00211-y

[CR13] Gibbons, O.P., Orr, J.J.: How to calculate embodied carbon (2022). https://www.egbc.ca/getmedia/a7603519-43cc-4795-8558-6960b2b7b5d1/HTCEC-2nd-edition.pdf

[CR14] Glas Trösch Wärmedämmisolierglas. Retrieved 28 Apr 2026 from https://www.glastroesch.com/ch/de/service/datentabellen/waermedaemmisolierglas

[CR15] Hartwell, R., Coult, G., Overend, M.: Mapping the flat glass value-chain: A material flow analysis and energy balance of UK production (2022). 10.21203/rs.3.rs-1401635/v1

[CR16] Hartwell, R., Overend, M.: Unlocking the Re-use Potential of Glass Façade Systems. In: Vitkala, J. (ed.) GPD Glass Performance Days, pp. 273–280. Tampere, Finland (2019)

[CR17] Honic, M., Kovacic, I., Aschenbrenner, P., Ragossnig, A.: Material passports for the end-of-life stage of buildings: challenges and potentials. J. Clean. Prod. **319**, 128702 (2021). 10.1016/j.jclepro.2021.128702

[CR18] ISO 9869-1. Thermal insulation—Building elements—In-situ measurement of thermal resistance and thermal transmittance—Part 1: Heat flow meter method (2014)

[CR19] Likins-White, M., Tenent, R.C., Zhai, Z.: Degradation of insulating glass units: thermal performance, measurements and energy impacts. Buildings **13**(2), 551 (2023). 10.3390/buildings13020551

[CR20] MEPLA Pro: Mepla Software GmbH. Darmstadt, Germany (2025)

[CR21] Niiranen, K., Säily, V.-P., Hartikainen, J.: Independent testing of Sparklike Laser™—non-destructive insulating glass gas fill analyser. Ce/papers **2**(5–6), 277–285 (2018). 10.1002/cepa.930

[CR22] Park, S., Kim, S.H., Jeong, H., Do, S.L., Kim, J.: In situ evaluation of the U-Value of a window using the infrared method. Energies **14**(7), 1904 (2021). 10.3390/en14071904

[CR23] Paschke, F., Bishara, N., Schulz, I., Kocer, C., Schneider, J., Maier, A.: In situ U g -value measurement on three different glazing types. J. Phys. Conf. Ser. **2069**, 012134 (2021). 10.1088/1742-6596/2069/1/012134

[CR24] Rose, A., Sack, N., Nothacker, K., Gassman, A.: Recycling of flat glass in the building industry—Analysis of the current situation and derivation of recommendations for action. ift Rosenheim (2019). Retrieved 12 Mar 2025 from https://www.irbnet.de/daten/kbf/kbf_e_F_3202.pdf

[CR25] Rota, A., Zaccaria, M., Fiorito, F.: Towards a quality protocol for enabling the reuse of post-consumer flat glass. Glass Struct. Eng. **8**(2), 235–254 (2023). 10.1007/s40940-023-00233-0

[CR26] Saint-Gobain Glass. Cool-Lite Xtreme ORAÉ - Performance meets sustainability: The world’s first low carbon glass ORAÉ combined with the best in class coatings. In: Product data sheet (2023)

[CR27] SIA 261. Actions on structures (2020)

[CR28] SIA 2057. Glass structures (2021)

[CR29] Silvestru, V.-A.: Experimental study on using thermal treatment for stress relief in thermally tempered glass. Glass Struct. Eng. **10**(4), 22 (2025). 10.1007/s40940-025-00308-0

[CR30] Snitzel, B., Steinke, G., Müller, K., Rudolf, B., Geissler, A., Blaser, R., Sibold, C., Messmer, C., Bender, J., Held, S.: FenSanReuse – Sanierungsverfahren und Re-Use von Fenstern – Materialpass und Wegleitung. Fachhochschule Nordwestschweiz (2025)

[CR31] Soares, N., Martins, C., Gonçalves, M., Santos, P., da Silva, L.S., Costa, J.J.: Laboratory and in-situ non-destructive methods to evaluate the thermal transmittance and behavior of walls, windows, and construction elements with innovative materials: a review. Energy Build. **182**, 88–110 (2019). 10.1016/j.enbuild.2018.10.021

[CR32] Teich, M., Scherer, C., Schuster, M., Brandenstein, M., Elstner, M.: Reuse and remanufacturing of insulated glass units. Glass Struct. Eng. **9**(3), 339–356 (2024). 10.1007/s40940-024-00276-x

[CR33] Tishman Speyer. Glass from glass—Leading by example: Setting a precedent in the construction industry (2018). Retrieved 11 Feb 2026 from https://ukgbc.s3.eu-west-2.amazonaws.com/wp-content/uploads/2018/09/05151714/VerdeSW1CaseStudy_FINALISSUE1.pdf

[CR34] United Nations. Paris Agreement (2015). Retrieved 06 Feb 2026 from https://unfccc.int/sites/default/files/english_paris_agreement.pdf

[CR35] Van Den Bergh, S., Hart, R., Jelle, B.P., Gustavsen, A.: Window spacers and edge seals in insulating glass units: a state-of-the-art review and future perspectives. Energy Build. **58**, 263–280 (2013). 10.1016/j.enbuild.2012.10.006

[CR36] van Nieuwenhuijzen, E.J., Tetteroo, J.I.A., van de Vliet, M., Melet, E.: In situ detection of product age and argon concentration as measure of the re-use potential of insulating glass units in buildings. Glass Struct. Eng. **8**(2), 211–233 (2023). 10.1007/s40940-023-00225-0

[CR37] Wolf, A.T.: Studies into the life-expectancy of insulating glass units. Build. Environ. **27**(3), 305–319 (1992). 10.1016/0360-1323(92)90032-K

[CR38] Wolf, A.T., Waters, L.J.: Factors governing the life expectancy of dual-sealed insulating glass units. Constr. Build. Mater. **7**(2), 101–107 (1993). 10.1016/0950-0618(93)90039-F

